# Antioxidative Indenone and Benzophenone Derivatives from the Mangrove-Derived Fungus *Cytospora heveae* NSHSJ-2

**DOI:** 10.3390/md21030181

**Published:** 2023-03-14

**Authors:** Ge Zou, Taobo Li, Wencong Yang, Bing Sun, Yan Chen, Bo Wang, Yanghui Ou, Huijuan Yu, Zhigang She

**Affiliations:** 1 School of Chemistry, Sun Yat-Sen University, Guangzhou 510275, China; 2 Guangdong Key Laboratory of Animal Conservation and Resource Utilization, Guangdong Public Laboratory of Wild Animal Conservation and Utilization, Institute of Zoology, Guangdong Academy of Sciences, Guangzhou 510260, China

**Keywords:** mangrove endophytic fungus, *Cytospora heveae*, indenone, benzophenone, DPPH·scavenging activity

## Abstract

Seven new polyketides, including four indenone derivatives, cytoindenones A–C (**1**, **3**–**4**), 3′-methoxycytoindenone A (**2**), a benzophenone derivative, cytorhizophin J (**6**), and a pair of tetralone enantiomers, (±)-4,6-dihydroxy-5-methoxy-*α*-tetralone (**7**), together with a known compound (**5**) were obtained from the endophytic fungus *Cytospora heveae* NSHSJ-2 isolated from the fresh stem of the mangrove plant *Sonneratia caseolaris*. Compound **3** represented the first natural indenone monomer substituted by two benzene moieties at C-2 and C-3. Their structures were determined by the analysis of 1D and 2D NMR, as well as mass spectroscopic data, and the absolute configurations of (±)-**7** were determined on the basis of the observed specific rotation value compared with those of the tetralone derivatives previously reported. In bioactivity assays, compounds **1**, **4**–**6** showed potent DPPH· scavenging activities, with EC_50_ values ranging from 9.5 to 16.6 µM, better than the positive control ascorbic acid (21.9 µM); compounds **2**–**3** also exhibited DPPH· scavenging activities comparable to ascorbic acid.

## 1. Introduction

Indenones are characterized by a cyclopentenone ring fused with an aromatic benzene ring, providing a rigid bicyclic ring framework which enables the extensive evaluation of structure–activity relationship analysis of target therapeutic molecules [1], and indenone derivatives have been synthesized extensively for drug discovery [2,3,4,5]. The indenone moiety usually exists in natural products as a structural fragment or a small independent molecule [6,7,8,9,10,11], and 2,3-diaryl indenone analogues are rarely reported [12,13,14]. These compounds were considered to be dimers of benzophenone, xanthone, diphenyl ether moieties and indanone moieties, and there was no natural 2,3-diphenyl indenone monomer reported previously. Indenones have multiple bioactivities, including cytotoxicity, DPPH· scavenging activity, anti-inflammatory activity, anti-osteoporosis activity, human DNA dealkylation repair enzyme AlkBH3 inhibitory activity, and PPAR *γ* agonistic activity [2,3,4,5,8,13,14,15].

**Figure 1:**
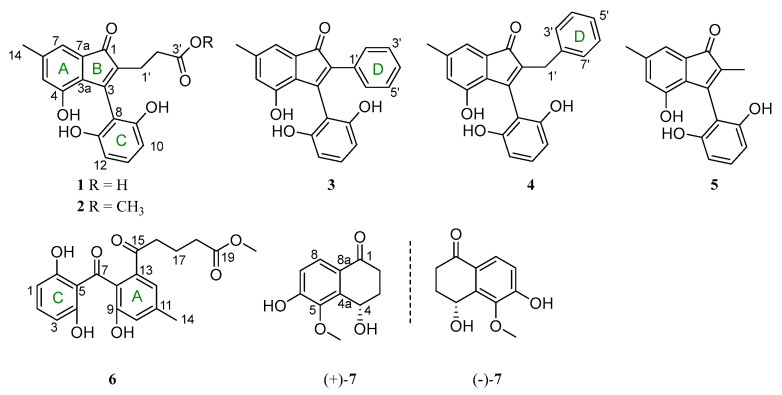
Structure of compounds **1**−**7**.

Mangrove-associated fungi are known to be an essential source of natural products for the discovery of new drug leads [16,17]. In our continuing search for structurally diverse and biologically active metabolites from mangrove endophytic fungi [18,19,20,21,22], a chemical investigation for new secondary metabolites from mangrove endophytic fungus *Cytospora heveae* NSHSJ-2, which was isolated from the fresh stem of the mangrove plant *Sonneratia caseolaris*, led to the isolation and characterization of seven new polyketides (Figure 1), including four new indenone derivatives, cytoindenones A–C (**1**, **3**–**4**), 3′-methoxycytoindenone A (**2**), a new benzophenone derivative, cytorhizophin J (**6**), and a pair of undescribed tetralone enantiomers, (±)-4,6-dihydroxy-5-methoxy-*α*-tetralone (**7**), together with a known compound, cytosporaphenones E (**5**) [23]. Among them, compound 3 represented the first natural indenone monomer substituted by two benzene moieties at C-2 and C-3. Herein, the isolation, structure elucidation, and DPPH· radical scavenging activities of these compounds are described.

## 2. Results

### 2.1. Structure Elucidation

Compound **1** was obtained as brown oil. Its molecular formula was assigned as C_19_H_16_O_6_ on the basis of HRESIMS analysis at *m/z* 363.08383 [M + Na]^+^ (calcd. For C_19_H_16_O_6_Na, 363.08391), which was determined to possess 12 degrees of unsaturation. In the ^1^H NMR spectrum (Table 1), the signals for five olefinic protons (*δ*_H_ 7.06, 6.79, 6.65, 6.50 and 6.50), two methylenes (*δ*_H_ 2.49 and 2.42) and one methyl (*δ*_H_ 2.24) were observed. The ^13^C NMR data (Table 2) exhibited 19 carbon resonances, including two carbonyls (*δ*_C_ 198.2 and 174.3), two aromatic rings (A and C) (*δ*_C_ 156.2, 156.2, 151.7, 140.7, 134.3, 130.7, 127.1, 124.5, 116.4, 110.6, 108.0, 108.0), two olefinic carbons for one double bond (*δ*_C_ 152.1, 134.4), two methylenes (*δ*_C_ 32.4 and 20.4) and one methyl (*δ*_C_ 21.0).

**Figure 2:**
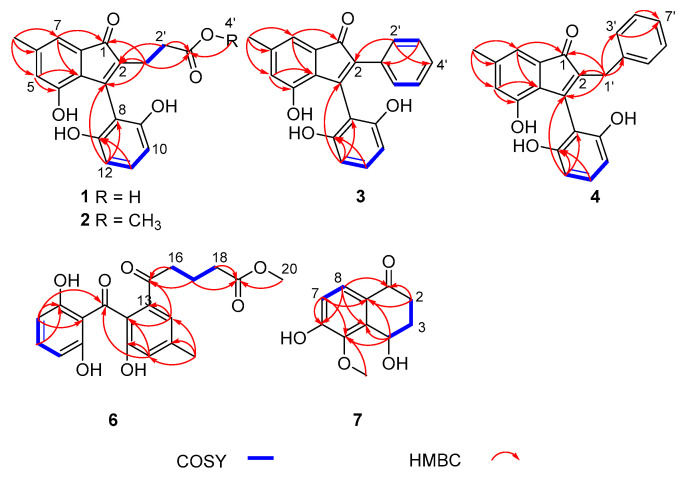
Key COSY and HMBC correlations of **1**–**4** and **6**–**7**.

The HMBC correlations from H-1′, to C-1, C-2, C-3, from H-14 to C-5, C-6, C-7, from H-5 to C-3a, C-4, and from H-7 to C-1, C-3a suggested the presence of an indenone fragment (rings A and B) (Figure 2). Additionally, the ^1^H-^1^H COSY correlations of H-10/H-11/H-12, together with the HMBC correlations from H-12 to C-3, C-8 and C-13, and from H-11 to C-13, completed the 2,6-dihydroxybenzoyl fragment (ring C), which connected to the indenone ring at C-3. The structures of ring A, B and C were further confirmed by comparison of ^1^H and ^13^C NMR spectra between **1** and **5** [23]. Furthermore, the ^1^H-^1^H COSY correlation of H-1′/H-2′ and the HMBC correlations from H-1′, H-2′ to C-3′, from H-1′ to C-1, C-2, C-3, and from H-2′, to C-2 indicated the presence of the 2-carboxyethyl group, which was assigned to be connected to the indenone ring at C-2. Thus, the structure of **1** was deduced, named cytoindenone A.

Compound **2** was isolated as brown oil. Its molecular formula was determined as C_20_H_18_O_6_ (12 degrees of unsaturation) in terms of HREIMS analysis at *m/z* 377.09985 [M + Na]^+^ (calcd. for C_20_H_18_O_6_Na, 377.09956). Analysis of the ^1^H and ^13^C NMR spectroscopic data of **2** (Table 1 and Table 2) revealed mostly similarities with that of **1**, except that the hydroxyl group was substituted with the methoxy group (*δ*_H_ 3.58, *δ*_C_ 52.2) at C-3′. Combined with the HMBC from H-4′ to C-3′ (Figure 2), the structure of compound **2** was clearly confirmed, named 3′-methoxycytoindenone A.

Compound **3** was acquired as brown oil and had a molecular formula of C_22_H_16_O_4_, determined by HRESIMS data *m/z* 367.09424 [M + Na]^+^ (calcd. 367.09408) with 15 degrees of unsaturation. The ^1^H NMR spectrum of **3** displayed the signal for ten olefinic protons (*δ*_H_ 7.30, 7.30, 7.15, 7.15, 7.13, 6.99, 6.87, 6.63, 6.33 and 6.33) and one methyl (*δ*_H_ 2.27). The ^13^C NMR data exhibited one carbonyl (*δ*_C_ 199.1), three aromatic rings (*δ*_C_ 156.5, 156.5, 153.1, 141.9, 134.4, 133.7, 130.7, 129.9, 129.9, 128.5, 128.5, 127.9, 127.1, 125.1, 116.9, 112.1, 107.9 and 107.9), two olefinic carbons for one double bond (*δ*_C_ 154.0, 133.6) and one methyl (*δ*_C_ 21.2) (Table 1 and Table 2). According to 1D NMR and 2D NMR data, the rings A, B and C of **2** were similar to that of **1**. The obvious difference was the absence of the 2-carboxyethyl group at the C-2 position of compound **1** and the presence of a phenyl group (ring D) at the C-2 position of compound **3**. Meanwhile, the ^1^H-^1^H COSY correlations of H-3′/H-4′/H-5′ failed to be identified because the chemical shifts of H-3′, H-4′ and H-5′ were overlapped; the ^1^H-^1^H COSY correlations of H-2′/H-3′, the HMBC from H-2′ to C-2, C-5′, and from H-3′ to C-1′ also indicated that ring D was formed and connected to the indenone ring at C-2, and the structure of compound **3** was determined, named cytoindenone B.

Compound **4** was obtained as brown oil. The molecular formula was determined as C_23_H_18_O_4_ on the basis of HRESIMS data at *m/z* 381.10980 [M + Na]^+^ (calcd. for C_23_H_18_O_4_Na, 381.10973), which was thus determined to possess 15 degrees of unsaturation. The ^1^H and ^13^C NMR spectroscopic data were listed in Table 1 and Table 2, which suggested that the structure of compound **4** was similar to compound **3**, except the presence of methylenes (*δ*_H_ 3.41, *δ*_C_ 30.5). Similarly, the ^1^H-^1^H COSY correlations of H-3′/H-4′/H-5′/H-6′/H-7′ failed to be identified because of the overlapping chemical shifts. Combined with the HMBC from H-1′ to C-1, C-2, C-3, C-2′, C-3′, and from H-3′, H-4′ to C-5′ (Figure 2), ring D was formed and C-1′ was connected to the indenone ring and ring D at C-2 and C-2′, and the structure of compound **5** was clearly confirmed, named cytoindenone C.

Compound **6** was isolated as white powder and assigned an HRESIMS ion peak at *m/z* 395.11005 ([M + Na]^+^, calcd. for C_20_H_20_O_7_Na, 395.11012), which perfectly matched the molecular formula of C_20_H_20_O_7_ with 11 degrees of unsaturation. The ^1^H NMR spectrum of **6** displayed the signal for five olefinic protons (*δ*_H_ 7.25, 7.18, 6.87, 6.27 and 6.27), one methoxyl (*δ*_H_ 3.63), three methylenes (*δ*_H_ 2.96, 2.32 and 1.85) and one methyl (*δ*_H_ 2.36). The ^13^C NMR data revealed 20 carbon resonances, involving two carbonyls (*δ*_C_ 204.1 and 202.2), one ester carbonyl (*d*_C_ 175.5), two aromatic rings (*δ*_C_ 163.2, 163.2, 155.0, 140.8, 137.8, 137.0, 130.0, 121.6, 121.6, 112.7, 108.1, 108.1), one methoxyl (*δ*_C_ 52.0), three methylenes (*δ*_C_ 38.9, 33.7 and 20.6) and one methyl (*δ*_C_ 21.3) (Table 3). According to 1D NMR and 2D NMR data, the benzophenone moiety of **6** was similar to cytorhizophin C [24]. The only difference between them were that the popionyl group at the C-13 position of cytorhizophin C was replaced by the 5-methoxy-5-oxopentanoyl group of compound **6**. The ^1^H-^1^H COSY correlations of H-16/H-17/H-18, together with the HMBC correlations from H-16 to C-13 and C-15, from H-17 to C-15, and from H-18, H-20 to C-19 indicated that the 5-methoxy-5-oxopentanoyl group was located at C-13. Therefore, the structure of **6** was deduced and named cytorhizophin J.

Compound **7** was acquired as colorless oil. Its molecular formula C_11_H_12_O_4_ (six degrees of unsaturation) was established on the basis of HREIMS analysis at *m/z* 209.08093 [M + H]^+^ (calcd. For C_11_H_13_O_4_, 209.08084). Analysis of the ^1^H and ^13^C NMR spectroscopic data of **7** (Table 4) revealed mostly similarities to 3,4-dihydro-4β,6-dihydroxy-5-methoxy-2*α*-methyl-1(2*H*)-naphthalenone [25]. The main difference between them were the absence of one methine at *δ*_H_ 2.98 (1H, m, H-2*β*) and one methyl at *δ*_H_ 1.11 (3H, d, *J* = 6.8 Hz, 2-Me) in 3,4-dihydro-4*β*,6-dihydroxy-5-methoxy-2*α*-methyl-1(2*H*)-naphthalenone and the presence of one methylene at *δ*_H_ 2.99 (1H, m, H_a_-2) and 2.43 (1H, dt, *J* = 17.2, 3.6, H_b_-2) in **7**, which was confirmed by the ^1^H-^1^H COSY correlations of H_a, b_-2/H_a, b_-3/H-4, and the HMBC correlations (Figure 2) from H_a, b_-2 to C-1, C-8a. Thus, compound **7** was assigned as shown in Figure 1, and named 4,6-dihydroxy-5-methoxy-*α*-tetralone. However, chiral HPLC analysis of **7** showed two peaks (*t*_R_ 21.3 min and 24.6 min), and subsequent chiral HPLC purification of (±)-**7** led to the separation of the two enantiomers (+)-**7** and (−)-**7**. The absolute configurations of (+)-**7** and (−)-**7** were determined as 4*S* and 4*R* by the comparison of the observed specific rotation value [(+)-**7**: ${\left[\alpha \right]}_{D}^{25}$ + 31.3, (+)-**7**: ${\left[\alpha \right]}_{D}^{25}$ − 31.5)] of compounds (±)-**7** with those for (4*S*)-4,8-dihydroxy-*α*-tetralone (${\left[\alpha \right]}_{D}^{27}$ + 24.5), (4*S*)-5-hydroxy-4-methoxy-*α*-tetralone (${\left[\alpha \right]}_{D}^{27}$ + 50.0), (4*R*)-4,8-dihydroxy-*α*-tetralone (${\left[\alpha \right]}_{D}^{27}$ − 26.0) and (4*R*)-5-hydroxy-4-methoxy-*α*-tetralone (${\left[\alpha \right]}_{D}^{27}$ − 50.0) (Figure S39) [26].

### 2.2. Biological Evaluation

Compounds **1**–**7** were tested for their DPPH· scavenging activity. As seen in Table 5, the results indicated that compounds **1**, **4**–**6** showed significant DPPH· scavenging activities with EC_50_ values ranging from 9.5 to 16.6 µM, better than the positive control ascorbic acid (21.9 µM) [27,28]; compounds **2**–**3** also exhibited DPPH· scavenging activities comparable to ascorbic acid.

The antioxidant activities of phenolic compounds were widely investigated and the phenolic content and the side chain functional groups had significant influences on DPPH· scavenging activities [29,30]. Comparing the activities of compounds **1**−**2**, when the carboxyl group at C-3′ was esterified by the methyl group, the antioxidant activity of **2** decreases significantly. Comparing the activities of compounds **2**−**5**, the higher activity of compound **5** was due to the accessibility of the phenolic OH group by DPPH·. The activities of compounds **2**−**4** were due to the presence of bulky groups at C-2 obstructing DPPH· access to the phenolic OH group. Compound **6** could be regarded as a precursor of compound **2**, which formed ring B through C7−C16 aldol-type cyclization. Compound **6** exhibited the strongest antioxidant activity due to the disconnection of ring B and the smallest steric hindrance of phenolic ring C. Compounds (+)-7 and (–)-7 showed no antioxidant activities due to the reduction of the phenolic content.

## 3. Experimental Section

### 3.1. General Experimental Procedures

Optical rotations were performed on an MCP300 (Anton Paar, Shanghai, China). UV data were measured on a Shimadzu UV-2600 spectrophotometer (Shimadzu, Kyoto, Japan). The ECD experiment data were conducted with a J-810 spectropolarimeter (JASCO, Tokyo, Japan). IR spectra were measured on an IR Affinity-1 spectrometer (Shimadzu, Kyoto, Japan). Melting points were recorded on a Fisher-Johns hot-stage apparatus. The NMR spectra were tested on a Bruker Avance spectrometer (Bruker, Beijing, China) (Compounds **3**–**4**: 600 MHz for ^1^H and 150 MHz for ^13^C; compounds **1**–**2** and **5**–**7**: 400 MHz for ^1^H and 100 MHz for ^13^C, respectively). HRESIMS data were conducted on a ThermoFisher LTQ-Orbitrap-LC-MS spectrometer (Palo Alto, CA, USA). Column chromatography (CC) was performed on silica gel (200–300 mesh, Marine Chemical Factory, Qingdao, China) and Sephadex LH-20 (Amersham Pharmacia, Piscataway, NJ, USA). Semi-preparative HPLC (Ultimate 3000 BioRS, Thermo Scientific, Germany) were conducted using the Chiral INA column (5 μm, 4.6 × 250 mm, Phenomenex, Piscataway, NJ, USA), and the Chiralcel ODH column (5 μm, 4.6 × 250 mm, Daicel, Tokyo, Japan) for chiral separation.

### 3.2. Fungal Material

The fungal strain NSHSJ-2 used in this study was isolated from the fresh stem of mangrove plant *Sonneratia caseolaris*, which was collected in June 2020 from the Nansha Mangrove National Nature Reserve in Guangdong Province, China. The strain was identified as *Cytospora heveae* (compared to no. OQ423127) upon the analysis of ITS sequence data of the rDNA gene. The ITS sequence data obtained from the fungal strain has been submitted to GenBank with accession no. OL780505.1. A voucher strain was deposited in our laboratory.

### 3.3. Fermentation, Extraction and Isolation

The fungus *Cytospora heveae* NSHSJ-2 was fermented on solid cultured medium (sixty 1000 mL Erlenmeyer flasks, each containing 50 g of rice and 50 mL of distilled water with 3% sea salt) for 30 days at 25 °C. The cultures were extracted three times with MeOH to yield 22.9 g of residue. Then, the crude extract was eluted by using a gradient elution with petroleum ether/EtOAc from 9:1 to 0:10 *(v/v*) on silica gel CC to get six fractions (Fr. A–F). Fr. D (297 mg) was subjected to silica gel CC (CH_2_Cl_2_/MeOH, *v/v*, 800:1 to 200:1) to obtain three subfractions (Fr. D_1_–D_3_). Fr. D_2_ (9.4 mg) was separated by normal phase HPLC on a chiral column (INA), using hexane/isopropanol (80:20, *v/v*, flow rate: 1.0 mL/min) as the solvent system, to yield compounds **3** (1.6 mg, *t*_R_ 14.0 min) and **4** (4.3 mg, *t*_R_ 21.2 min). Fr. D_3_ (83.4 mg) was applied to Sephadex LH-20 CC (CH_2_Cl_2_/MeOH, *v/v*, 1:1) to yield compound **5** (26 mg). Fr. E (749 mg) was subjected to silica gel CC (CH_2_Cl_2_/MeOH, *v/v*, 100:1 to 20:1) to afford four fractions (Fr. E_1_−E_4_). Fr. E_2_ (204 mg) was subjected to silica gel CC (petroleum ether/EtOAc, *v/v*, 7:3) to yield compounds **2** (46.5 mg). Fr. E_3_ (56.4 mg) was subjected to silica gel CC (petroleum ether/EtOAc, *v/v*, 6:4) to yield compounds **6** (15.4 mg) and (±)-**7** (9.4 mg). The chiral HPLC separation of (±)-**7** was accomplished over a chiral column (ODH) (column size: 4.6 × 250 mm, 5 μm; flow rate: 1.0 mL/min; solvent: n-hexane-isopropanol = 90:10) to yield (+)-**7** (1.4 mg, *t*_R_ 21.3 min) and (−)-**7** (7.3 mg, *t*_R_ 24.6 min). Fr. E_4_ (103 mg) was purified by Sephadex LH-20 CC and eluted with MeOH to obtain compound **1** (27.9 mg).

Cytoindenone A (**1**): brown oil; UV (MeOH) *λ*_max_ (log *ε*): 205 (1.24), 247 (0.53) nm; IR *υ*_max_ 3282, 2949, 2835, 1695, 1435, 1276, 1010, 781 cm^−1^; HRESIMS *m/z* 363.08383 [M + Na]^+^ (calcd. for C_19_H_16_O_6_Na, 363.08391); ^1^H NMR (400 MHz, Actone-*d*_6_) data and ^13^C NMR (100 MHz, Actone-*d*_6_) data (see Table 1 and Table 2).

3′-methoxycytoindenone A (**2**): brown oil; UV (MeOH) *λ*_max_ (log *ε*): 204 (0.90), 248 (0.42) nm; IR *υ*_max_ 3360, 2954, 2920, 1697, 1622, 1462, 1278, 1012, 783 cm^−1^; HRESIMS *m/z* 377.09985 [M + Na]^+^ (calcd. for C_20_H_18_O_6_Na, 377.09956); ^1^H NMR (400 MHz, CDCl_3_) data and ^13^C NMR (100 MHz, CDCl_3_) data (see Table 1 and Table 2).

Cytoindenone B (**3**): brown oil; UV (MeOH) *λ*_max_ (log *ε*): 203 (0.32), 272 (0.15) nm; IR *υ*_max_ 3365, 2949, 2850, 1689, 1618, 1462, 1280, 1014, 792 cm^−1^; HRESIMS *m/z* 367.09424 [M + Na]^+^ (calcd. for C_22_H_16_O_4_Na, 367.09408); ^1^H NMR (600 MHz, CD_3_OD) data and ^13^C NMR (150 MHz, CD_3_OD) data (see Table 1 and Table 2).

Cytoindenone C (**4**): brown oil; UV (MeOH) *λ*_max_ (log *ε*): 205 (0.80), 249 (0.35) nm; IR *υ*_max_ 3358, 2922, 2852, 1683, 1618, 1462, 1276, 1012, 700 cm^−1^; HRESIMS *m/z* 381.10980 [M + Na]^+^ (calcd. for C_23_H_18_O_4_Na, 381.10973); ^1^H NMR (600 MHz, CD_3_OD) data and ^13^C NMR (150 MHz, CD_3_OD) data (see Table 1 and Table 2).

Cytorhizophin J (**6**): white powder, mp 190.2−191.6 ◦C; UV (MeOH) *λ*_max_ (log *ε*): 216 (1.43), 270 (0.67) nm; IR *υ*_max_ 3342, 2924, 1716, 1627, 1456, 1338, 1226, 1031, 925, cm^−1^; HRESIMS *m/z* 395.11005 [M + Na]^+^ (calcd. for C_20_H_20_O_7_Na, 395.11012); ^1^H NMR (400 MHz, CD_3_OD) and ^13^C NMR (100 MHz, CD_3_OD) data (see Table 3).

(±)-4,6-dihydroxy-5-methoxy-*α*-tetralone (**7**): colorless oil; UV (MeOH) *λ*_max_ (log *ε*): 205 (1.63), 230 (1.29), 282 (1.01) nm; IR *ν*_max_ 3261, 2943, 2839, 1660, 1578, 1305, 1290, 1190, 1012 cm^−1^; ^1^H NMR (400 MHz, CD_3_OD) data and ^13^C NMR (100 MHz, CD_3_OD) data (see Table 4); HRESIMS *m/z* 209.08093 [M + H]^+^ (calcd for C_11_H_13_O_4_, 209.08084). (+)-**7**, ${\left[\alpha \right]}_{D}^{25}$+ 31.3 (*c* 0.1 MeOH); ECD (*c* = 0.18 mg/mL, MeOH) *λ*_max_ (Δ*ε*) 205 (+13.5), 230 (+8.4), 284 (+7.0), 327 (−6.0). (−)-**7**, ${\left[\alpha \right]}_{D}^{25}$− 31.5 (c 0.1 MeOH); ECD (*c* = 0.17 mg/mL, MeOH) *λ*_max_ (Δ*ε*) 205 (−14.9), 225 (−6.1), 277 (−5.4), 320 (+5.8).

### 3.4. Biological Assays

The DPPH·radical scavenging activities of compounds **1**−**7** were determined according to the reported method [14,28]. The DPPH· radical scavenging test was performed in 96-well microplates. Testing materials (compounds **1**–**7**) were added to 100 µL (0.16 mmol/L) DPPH solution in MeOH at a range of 100 µL solutions of different concentrations (6.25−100 µM). Ascorbic acid was prepared as positive control at the same concentrations (Table 5). Absorbance was recorded at λ = 517 nm after 45 min of incubation in the dark. The DPPH·radical scavenging activity was calculated using the formula:

DPPH radical scavenging activity (%) = [(Abs_control_ − Abs_sample_)/Abs_control_] × 100

## 4. Conclusions

In summary, seven new polyketides including four indenone derivatives, cytoindenones A–C (**1**, **3**–**4**), 3′-methoxycytoindenone A (**2**), a new benzophenone derivative, cytorhizophin J (**6**) and a pair of undescribed tetralone enantiomers, (±)-4,6-dihydroxy-5-methoxy-1-tetralone (**7**), together with a known compound (**5**), were isolated from the endophytic fungus *Cytospora heveae* NSHSJ-2. Compound 3 represented the first natural indenone monomer substituted by two benzene moieties at C-2 and C-3. Their structures were confirmed by the analysis of NMR, HR-MS and ECD spectra. All of the compounds were tested for their antioxidative activities. Compounds **1**, **4**–**6** showed potent DPPH· scavenging activities with EC_50_ values ranging from 9.5 to 16.6 µM, better than the positive control ascorbic acid (21.9 µM); compounds **2**–**3** also exhibited DPPH· scavenging activities comparable to ascorbic acid.

## Figures and Tables

**Table 1:** ^1^H NMR data of **1**−**4** (*J* in Hz).

No.	1 ^a^	2 ^b^	3 ^c^	4 ^c^
5	6.65, s	6.54, s	6.63, s	6.58, s
7	6.79, s	6.82, s	6.87, s	6.75, s
10	6.50, d (8.1)	6.57, d (8.3)	6.33, d (8.1)	6.40, d (8.2)
11	7.06, t, (8.1)	7.15, t (8.3)	6.99, t (8.1)	7.02, t (8.2)
12	6.50, d (8.1)	6.57, d (8.3)	6.33, d (8.1)	6.40, d (8.2)
14	2.24, s	2.17, s	2.27, s	2.22, s
1′	2.42, m	2.42, t (7.1)		3.41, s
2′	2.49, m	2.66, t (7.1)	7.30, m	
3′			7.15, m	7.09, m
4′		3.58, s	7.13, m	7.07, m
5′			7.15, m	7.02, t (8.2)
6′			7.30, m	7.07, m
7′				7.09, m

^a^ Data were recorded in Actone-*d*_6_ at 400 MHz for ^1^H NMR. ^b^ Data were recorded in CDCl_3_ at 400 MHz for ^1^H NMR. ^c^ Data were recorded in CD_3_OD at 600 MHz for ^1^H NMR.

**Table 2:** ^13^C NMR data of **1**−**4**.

No.	1 ^a^	2 ^b^	3 ^c^	4 ^c^
1	198.2, C	197.5, C	199.1, C	200.2, C
2	134.4, C	135.2, C	133.6, C	134.8, C
3	152.1, C	148.3, C	154.0, C	154.8, C
3a	127.1, C	123.7, C	127.1, C	127.4, C
4	151.7, C	150.0, C	153.1, C	152.3, C
5	124.5, CH	124.7, CH	125.1, CH	125.0, CH
6	140.7, C	141.5, C	141.9, C	141.1, C
7	116.4, CH	118.2, CH	116.9, CH	116.6, CH
7a	134.3, C	132.5, C	134.4, C	134.6, C
8	110.6, C	108.4, C	112.1, C	111.4, C
9	156.2, C	153.6, C	156.5, C	156.6, C
10	108.0, CH	109.4, CH	107.9, CH	107.8, CH
11	130.7, CH	131.9, CH	130.7, CH	130.8, CH
12	108.0, CH	109.4, CH	107.9, CH	107.8, CH
13	156.2, C	153.6, C	156.5, C	156.6, C
14	21.0, CH_3_	21.3, CH_3_	21.2, CH_3_	21.1, CH_3_
1′	20.4, CH_2_	19.6, CH_2_	133.7, C	30.5, CH_2_
2′	32.4, CH_2_	31.0, CH_2_	129.9, CH	140.9, C
3′	174.3, C	175.1, C	128.5, CH	129.7, CH
4′		52.2, CH_3_	127.9, CH	128.9, CH
5′			128.5, CH	126.5, CH
6′			129.9, CH	128.9, CH
7′				129.7, CH

^a^ Data were recorded in Actone-*d*_6_ at 100 MHz for ^13^C NMR. ^b^ Data were recorded in CDCl_3_ at 100 MHz for ^13^C NMR. ^c^ Data were recorded in CD_3_OD at 150 MHz for ^13^C NMR.

**Table 3:** ^1^H and ^13^C NMR data for **6**.

	6 ^a^	
No.	*δ*_C_, Type	*δ*_H_ Mult (*J* in Hz)
1	108.1, CH	6.27, d (8.2)
2	137.0, CH	7.18, t (8.2)
3	108.1, CH	6.27, d (8.2)
4	163.2, C	
5	112.7, C	
6	163.2, C	
7	204.1, C	
8	130.0, C	
9	155.0, C	
10	121.6, CH	6.87, s
11	140.8, C	
12	121.6, CH	7.25, s
13	137.8, C	
14	21.3, CH_3_	2.36, s
15	202.2, C	
16	38.9, CH_2_	2.96, t (7.5)
17	20.6, CH_2_	1.85, m
18	33.7, CH_2_	2.32, t (7.3)
19	175.5, C	
20	52.0, CH_3_	3.63, s

^a^ Data were recorded in CD_3_OD at 400 MHz for ^1^H NMR and 100 MHz for ^13^C NMR.

**Table 4:** ^1^H and ^13^C NMR data for **7**.

	7 ^a^	
No.	*δ*_C_, Type	*δ*_H_ Mult (*J* in Hz)
1	199.6, C	
2	33.0, CH_2_	2.99, m
		2.43, dt (17.2, 3.6)
3	31.6, CH_2_	2.26, m
		2.17, m
4	61.7, CH	5.26, t (3.1)
4a	139.9, C	
5	146.2, C	
6	157.5, C	
7	117.8, C	6.92, d (8.6)
8	125.4, CH	7.67, d (8.6)
8a	125.6, C	
9	61.9, CH_3_	

^a^ Data were recorded in CD_3_OD at 400 MHz for ^1^H NMR and 100 MHz for ^13^C NMR.

**Table 5:** DPPH· scavenging activities of compounds **1**–**9**.

Compound	% Inhibition (100 µM)	EC_50_ (µM)
**1**	90.8	11.5 ± 0.1
**2**	72.5	21.5 ± 1.0
**3**	69.0	19.7 ± 1.8
**4**	78.2	16.6 ± 0.4
**5**	81.0	12.4 ± 0.5
**6**	87.3	9.5 ± 0.1
(+)-**7**	12.0	–
(–)-**7**	4.2	–
ascorbic acid ^a^	91.4	21.9 ± 0.3

^a^ positive control.

## Data Availability

Data are contained within the article and Supplementary Material.

## Supplementary Materials

The following are available online at https://www.mdpi.com/article/10.3390/md21030181/s1, Figure S1: HRESIMS spectrum of compound **1**; Figure S2: ^1^H NMR spectrum of compound **1** (400 MHz, Actone-*d*_6_); Figure S3: ^13^C NMR spectrum of compound **1** (100 MHz, Actone-*d*_6_); Figure S4: ^1^H-^1^H COSY spectrum of compound **1**; Figure S5: HSQC spectrum of compound **1**; Figure S6: HMBC spectrum of compound **1**; Figure S7: HRESIMS spectrum of compound **2**; Figure S8: ^1^H NMR spectrum of compound **2** (400 MHz, CDCl_3_); Figure S9: ^13^C NMR spectrum of compound **2** (100 MHz, CDCl_3_); Figure S10: ^1^H-^1^H COSY spectrum of compound **2**; Figure S11: HSQC spectrum of compound **2**; Figure S12: HMBC spectrum of compound **2**; Figure S13: HRESIMS spectrum of compound **3**; Figure S14: ^1^H NMR spectrum of compound **3** (600 MHz, CD_3_OD); Figure S15: ^13^C NMR spectrum of compound **3** (150 MHz, CD_3_OD); Figure S16: ^1^H-^1^H COSY spectrum of compound **3**; Figure S17: HSQC spectrum of compound **3**; Figure S18: HMBC spectrum of compound **3**; Figure S19: HRESIMS spectrum of compound **4**; Figure S20: ^1^H NMR spectrum of compound **4** (600 MHz, CD_3_OD); Figure S21: ^13^C NMR spectrum of compound **4** (150 MHz, CD_3_OD); Figure S22: ^1^H-^1^H COSY spectrum of compound **4**; Figure S23: HSQC spectrum of compound **4**; Figure S24: HMBC spectrum of compound **4**; Figure S25: HRESIMS spectrum of compound **6**; Figure S26: ^1^H NMR spectrum of compound **6** (400 MHz, CD_3_OD); Figure S27: ^13^C NMR spectrum of compound **6** (100 MHz, CD_3_OD); Figure S28: ^1^H-^1^H COSY spectrum of compound **6**; Figure S29: HSQC spectrum of compound **6**; Figure S30: HMBC spectrum of compound **6**; Figure S31: HRESIMS spectrum of compound **7**; Figure S32: ^1^H NMR spectrum of compound **7** (400 MHz, CD_3_OD); Figure S33: ^13^C NMR spectrum of compound **7** (100 MHz, CD_3_OD); Figure S34: ^1^H-^1^H COSY spectrum of compound **7**; Figure S35: HSQC spectrum of compound **7**; Figure S36: HMBC spectrum of compound **7**; Figure S37: ECD spectrum of compound (+)-7; Figure S38: ECD spectrum of compound (–)-7; Figure S39: Structure of compounds 3,4-dihydro-4*β*,6-dihydroxy-5-methoxy-2*α*-methyl-1(2*H*)-naphthalenone, (4*S*)-4,8-dihydroxy-α-tetralone, (4*R*)-4,8-dihydroxy-*α*-tetralone, (4*S*)-5-hydroxy-4-methoxy-α-tetralone and (4*R*)- 5-hydroxy-4-methoxy-*α*-tetralone.
