# Supplementary material for: Antioxidative Indenone and Benzophenone Derivatives from the Mangrove-Derived Fungus Cytospora heveae NSHSJ-2
Source: Mar Drugs. 2023 Mar 14;21(3):181. doi: 10.3390/md21030181 (PMC10057025; doi:10.3390/md21030181)
Supplement: Supplementary file 1 [file marinedrugs-21-00181-s001.zip › marinedrugs-2271584-supplementary-3.15.pdf]

Supporting information

Antioxidative indenone and benzophenone derivatives from the Mangrove-Derived Fungus *Cytospora heveae* NSHSJ-2

Ge Zou [1], Taobo Li [1], Wencong Yang [1], Bing Sun [1], Yan Chen [1], Bo Wang [1], Yanghui Ou [2], Huijuan Yu [2,*], and Zhigang She [1,*]

1 School of Chemistry, Sun Yat-Sen University, Guangzhou 510275, China;

2 Guangdong Key Laboratory of Animal Conservation and Resource Utilization, Guangdong Public Laboratory of Wild Animal Conservation and Utilization, Institute of Zoology, Guangdong Academy of Sciences, Guangzhou 510260, China.

* Correspondence: yuhj@giz.gd.cn(H.Y); cesshzhg@mail.sysu.edu.cn (Z.S.)

Supporting information

Figure S1. HRESIMS spectrum of compound **1**.

Figure S2. ^{1}H NMR spectrum of compound **1** (400 MHz, Actone-d_6).

Figure S3. ^{13}C NMR spectrum of compound **1** (100 MHz, Actone-d_6).

Figure S4. ^{1}H-^{1}H COSY spectrum of compound **1**.

Figure S5. HSQC spectrum of compound **1**.

Figure S6. HMBC spectrum of compound **1**.

Figure S7. HRESIMS spectrum of compound **2**.

Figure S8. ^{1}H NMR spectrum of compound **2** (400 MHz, $CDCl_3$).

Figure S9. ^{13}C NMR spectrum of compound **2** (100 MHz, $CDCl_3$).

Figure S10. ^{1}H-^{1}H COSY spectrum of compound **2**.

Figure S11. HSQC spectrum of compound **2**.

Figure S12. HMBC spectrum of compound **2**.

Figure S13. HRESIMS spectrum of compound **3**.

Figure S14. ^{1}H NMR spectrum of compound **3** (600 MHz, CD_3OD).

Figure S15. ^{13}C NMR spectrum of compound **3** (150 MHz, CD_3OD).

Figure S16. ^{1}H-^{1}H COSY spectrum of compound **3**.

Figure S17. HSQC spectrum of compound **3**.

Figure S18. HMBC spectrum of compound **3**.

Figure S19. HRESIMS spectrum of compound **4**.

Figure S20. ^{1}H NMR spectrum of compound **4** (600 MHz, CD_3OD).

Figure S21. ^{13}C NMR spectrum of compound **4** (150 MHz, CD_3OD).

Figure S22. ^{1}H-^{1}H COSY spectrum of compound **4**.

Figure S23. HSQC spectrum of compound **4**.

Figure S24. HMBC spectrum of compound **4**.

Figure S25. HRESIMS spectrum of compound **6**.

Figure S26. ^{1}H NMR spectrum of compound **6** (400 MHz, CD_3OD).

Figure S27. ^{13}C NMR spectrum of compound **6** (100 MHz, CD_3OD).

Figure S28. ^{1}H-^{1}H COSY spectrum of compound **6**.

Figure S29. HSQC spectrum of compound **6**.

Figure S30. HMBC spectrum of compound **6**.

Figure S31. HRESIMS spectrum of compound **7**.

Figure S32. ^{1}H NMR spectrum of compound **7** (400 MHz, CD_3OD).

Figure S33. ^{13}C NMR spectrum of compound **7** (100 MHz, CD_3OD).

Figure S34. ^{1}H-^{1}H COSY spectrum of compound **7**.

Figure S35. HSQC spectrum of compound **7**.

Figure S36. HMBC spectrum of compound **7**.

Figure S37. ECD spectrum of compound (+)-**7**.

Figure S38. ECD spectrum of compound (–)-**7**.

Figure S39. Structure of compounds 3,4-dihydro-4*β*,6-dihydroxy-5-methoxy-2*α*-methyl-1(2*H*)-naphthalenone, (4*S*)-4,8-dihydroxy-*α*-tetralone, (4*R*)-4,8-dihydroxy-*α*-tetralone, (4*S*)-5-hydroxy-4-methoxy-*α*-tetralone and (4*R*)- 5-hydroxy-4-methoxy-*α*-tetralone.

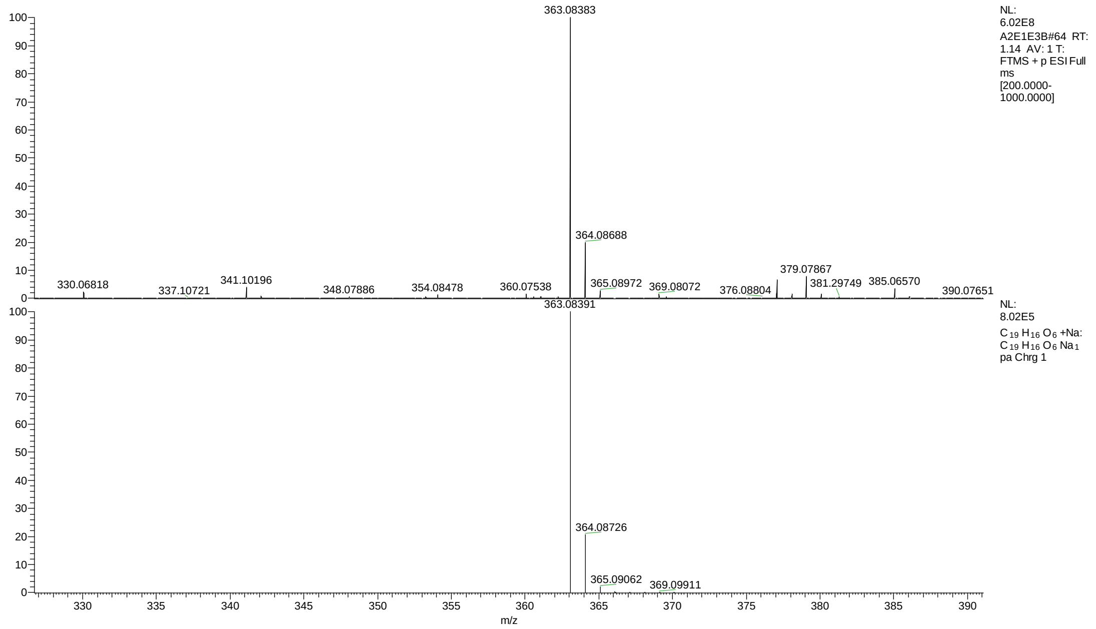

Figure S1. HRESIMS spectrum of compound **1**.

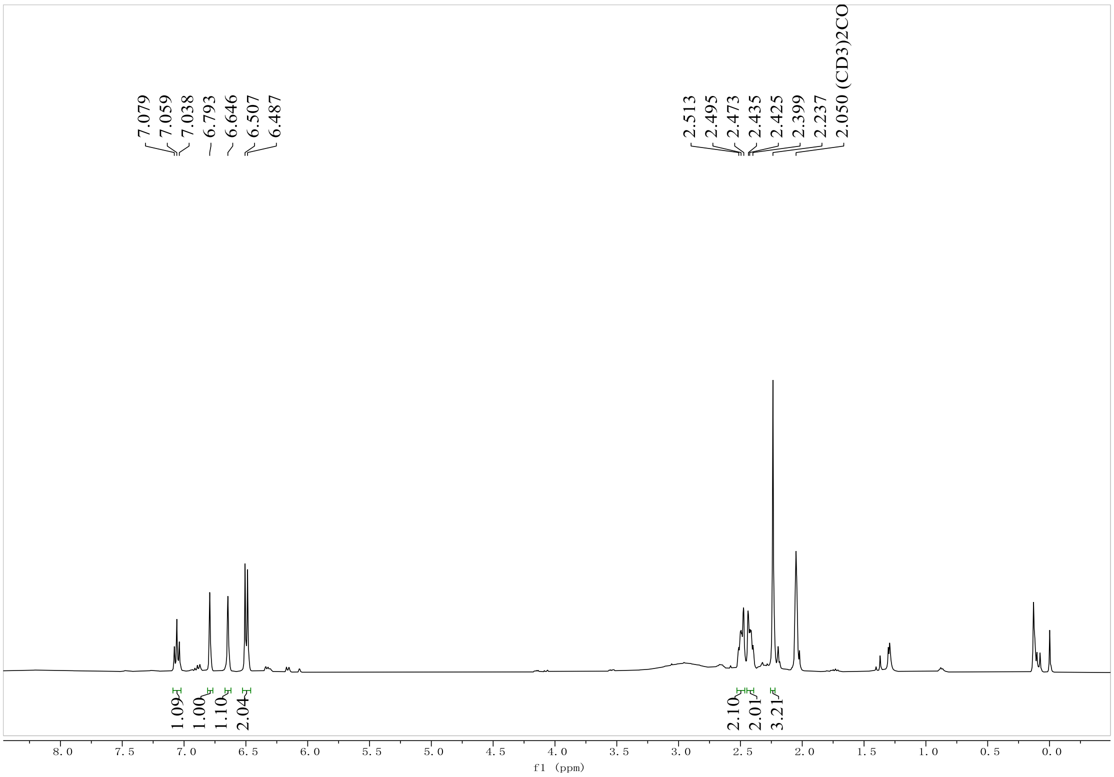

Figure S2. ^{1}H NMR spectrum of compound **1** (400 MHz, Actone-d_6).

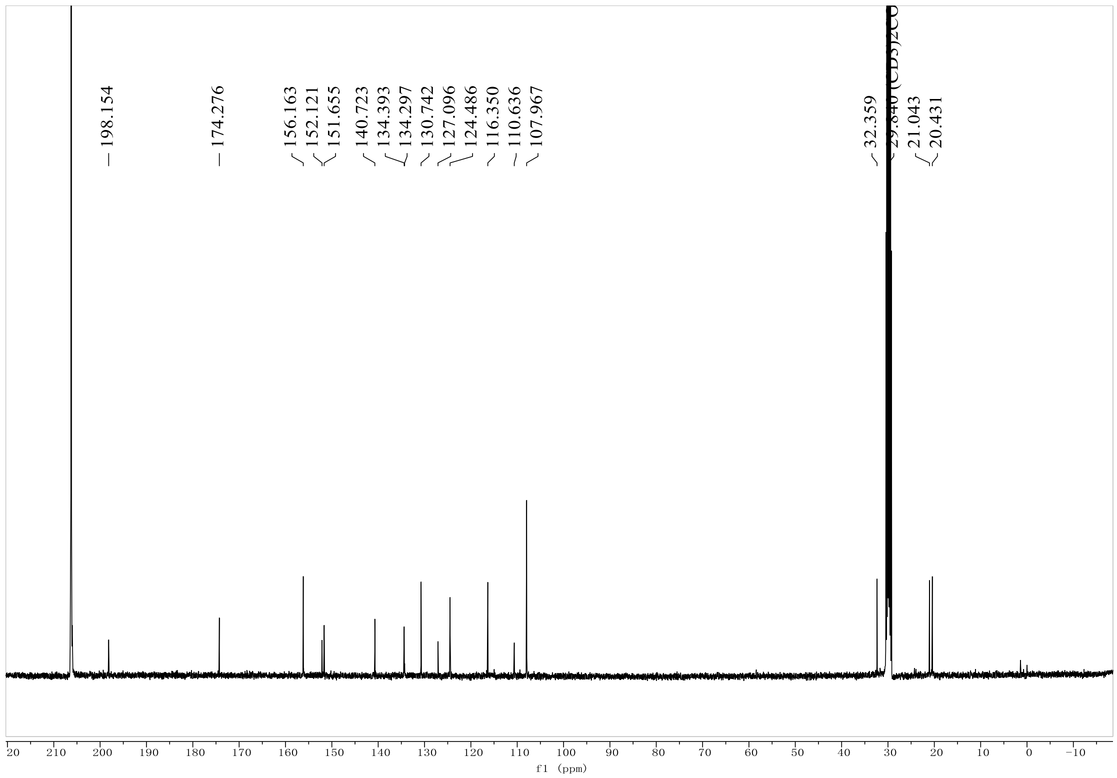

Figure S3. ^{13}C NMR spectrum of compound **1** (100 MHz, Actone-d_6).

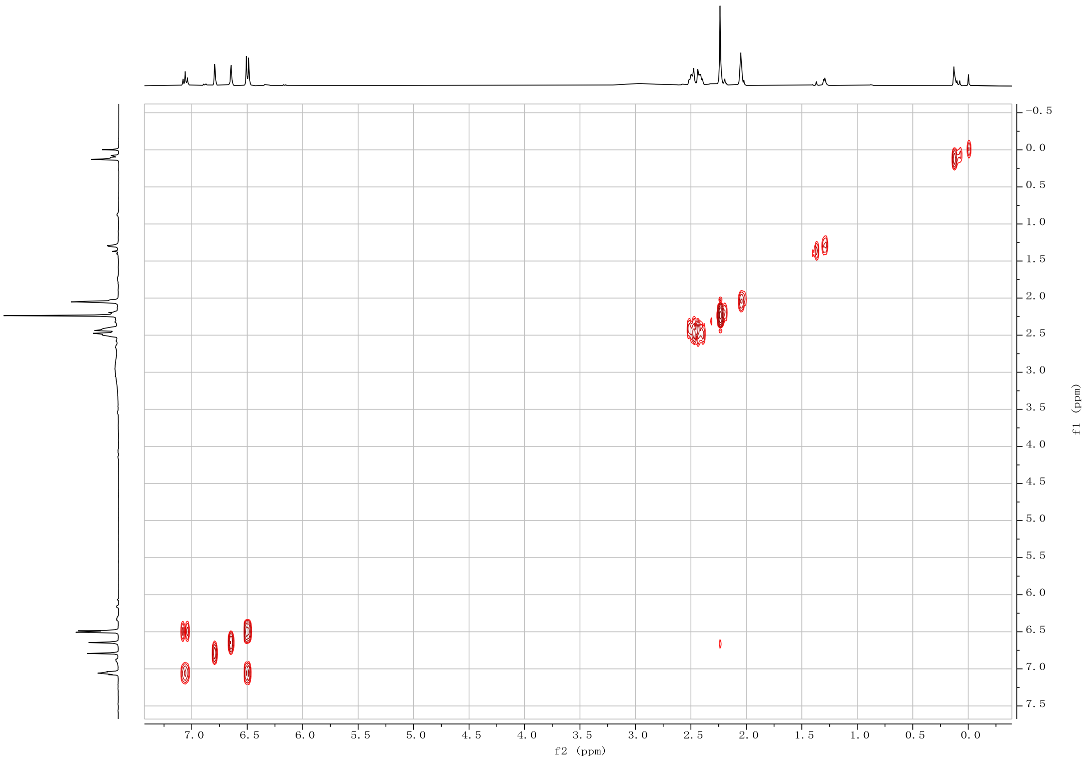

Figure S4. ^{1}H-^{1}H COSY spectrum of compound **1**.

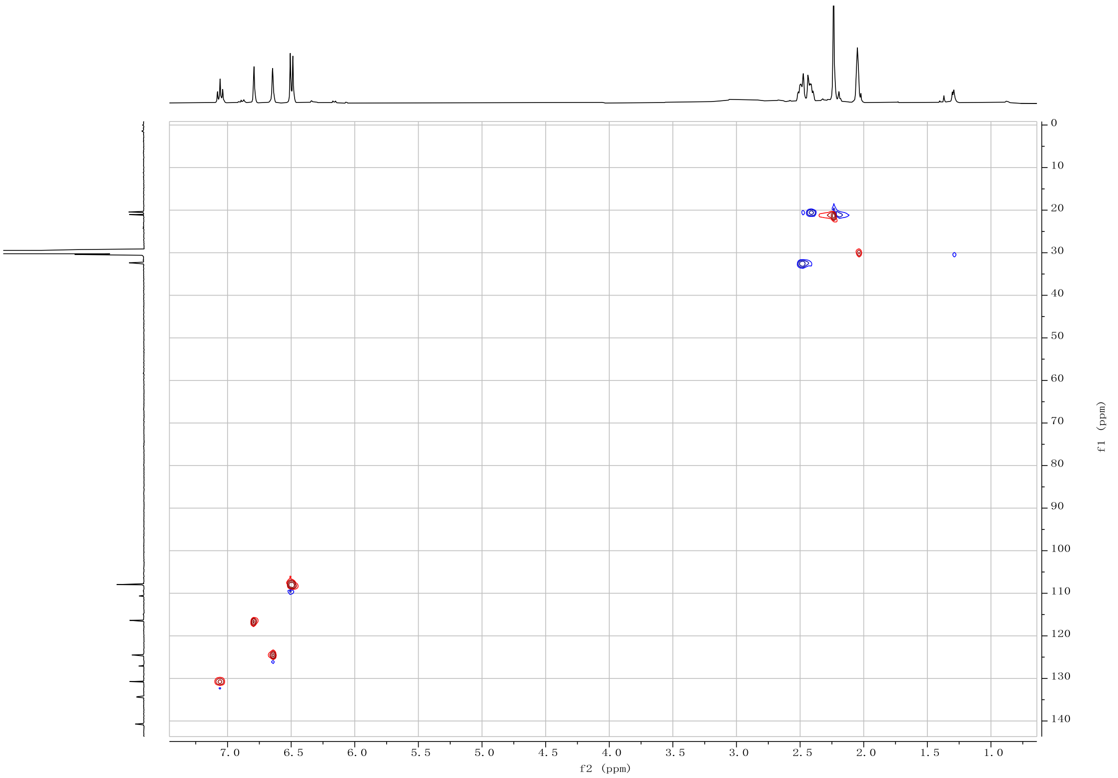

Figure S5. HSQC spectrum of compound **1**.

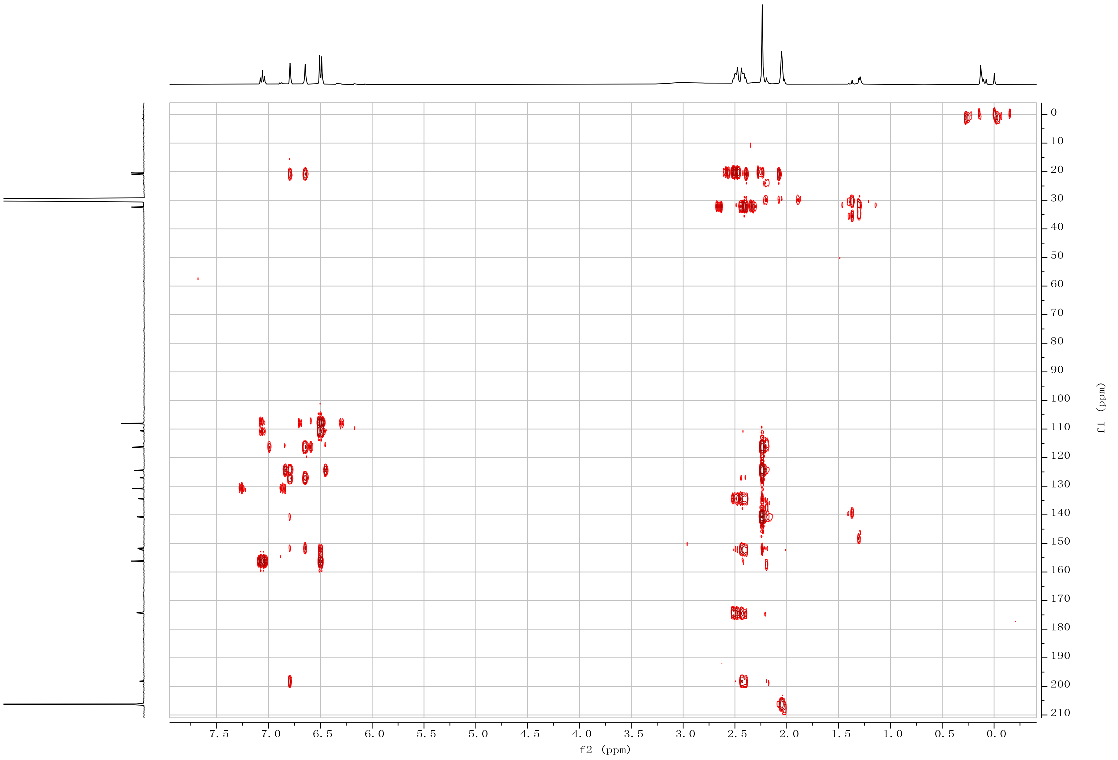

Figure S6. HMBC spectrum of compound **1**.

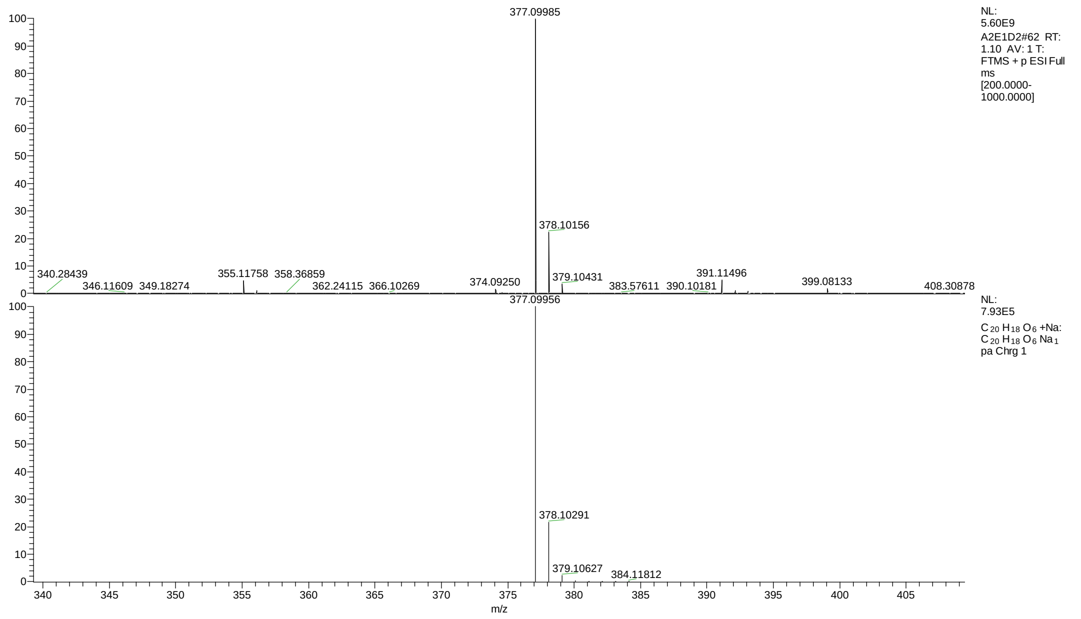

Figure S7. HRESIMS spectrum of compound **2**.

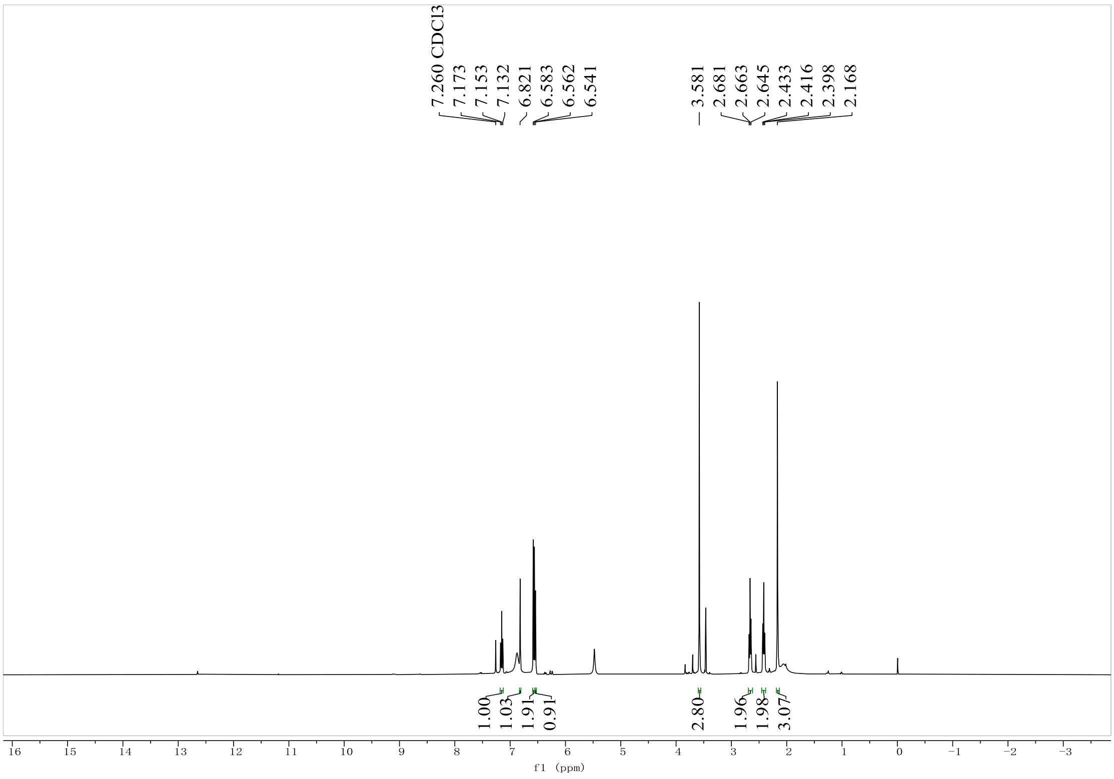

Figure S8. ^{1}H NMR spectrum of compound **2** (400 MHz, $CDCl_3$).

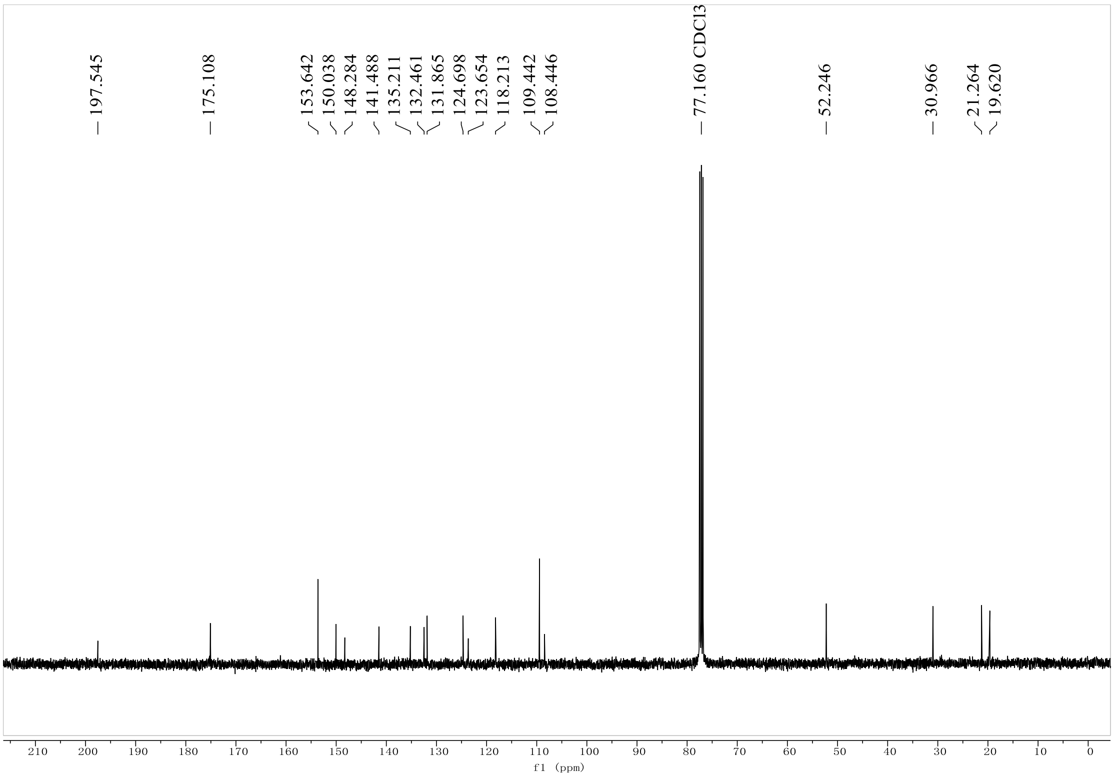

Figure S9. ^{13}C NMR spectrum of compound **2** (100 MHz, $CDCl_3$).

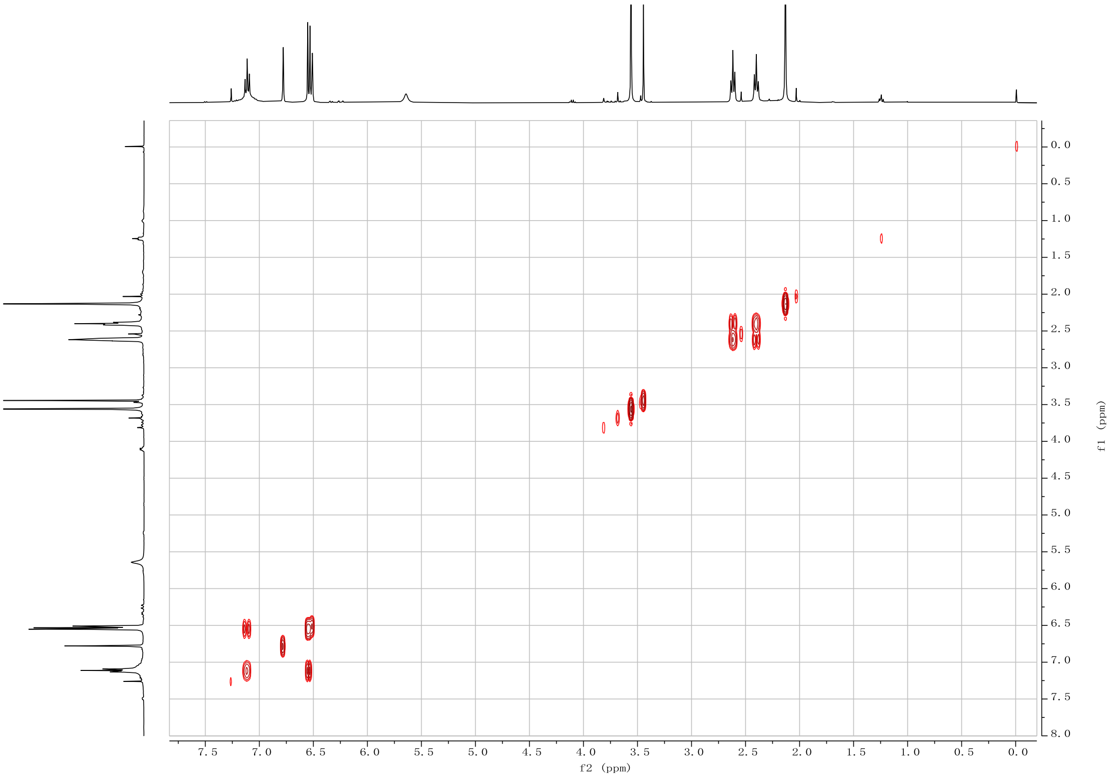

Figure S10. ^{1}H-^{1}H COSY spectrum of compound **2**.

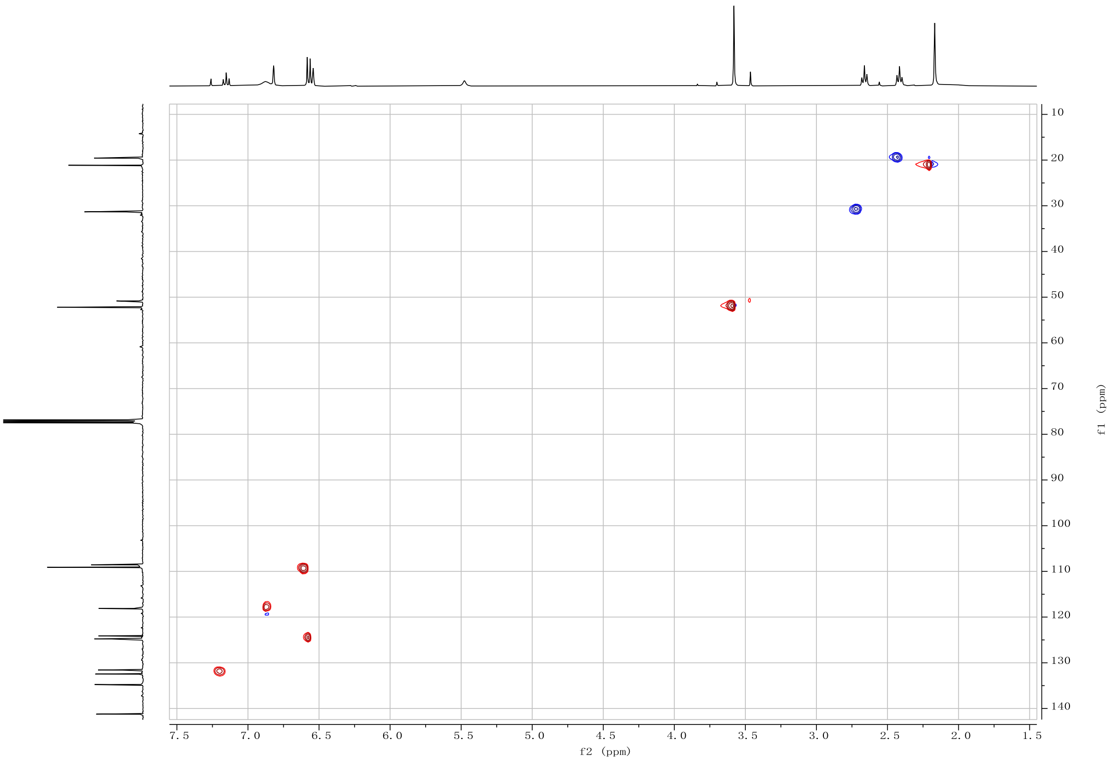

Figure S11. HSQC spectrum of compound **2**.

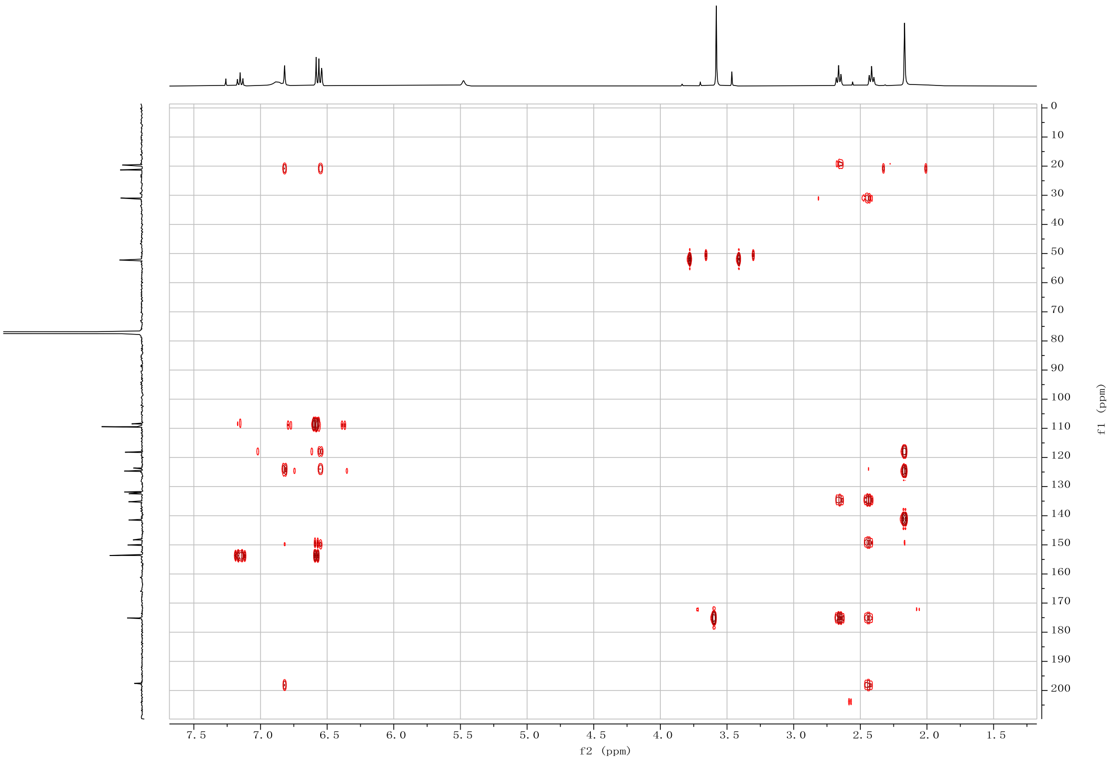

Figure S12. HMBC spectrum of compound **2**.

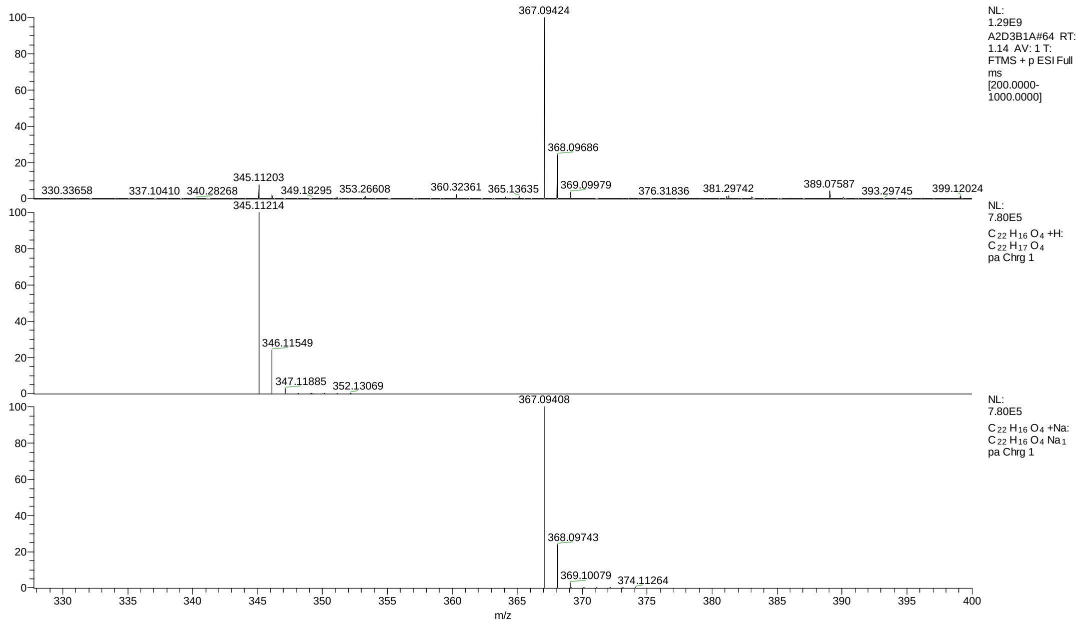

Figure S13. HRESIMS spectrum of compound **3**.

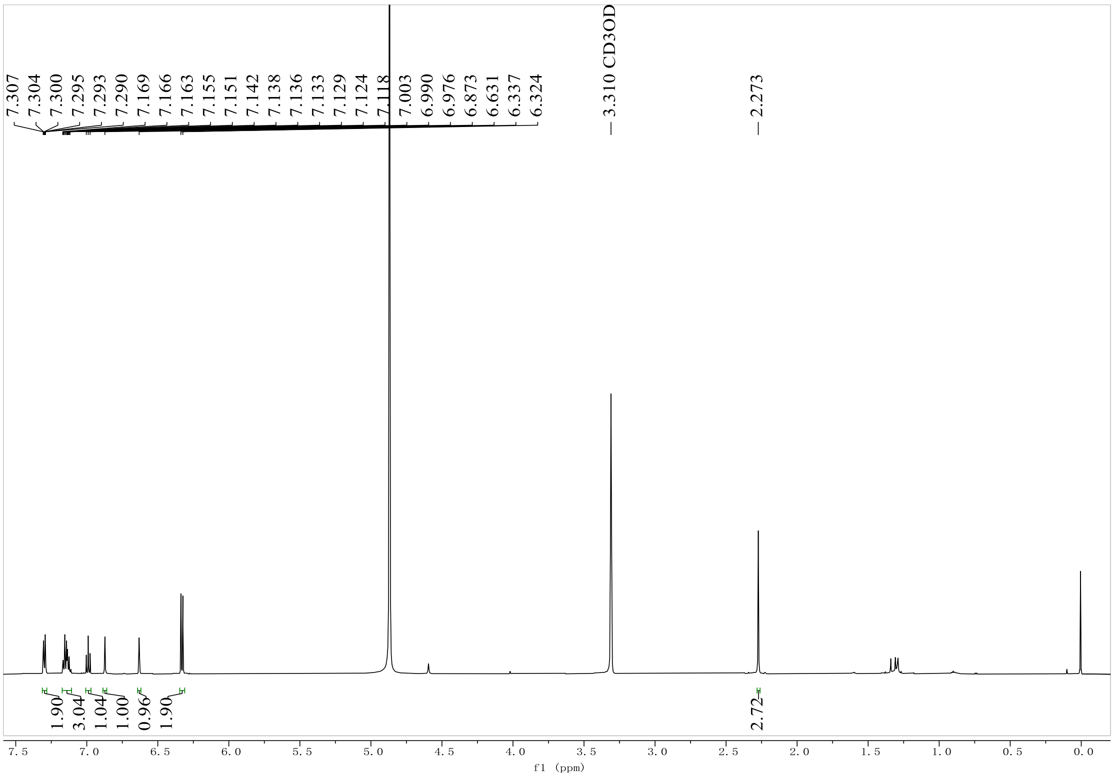

Figure S14. ^{1}H NMR spectrum of compound **3** (600 MHz, CD_3OD).

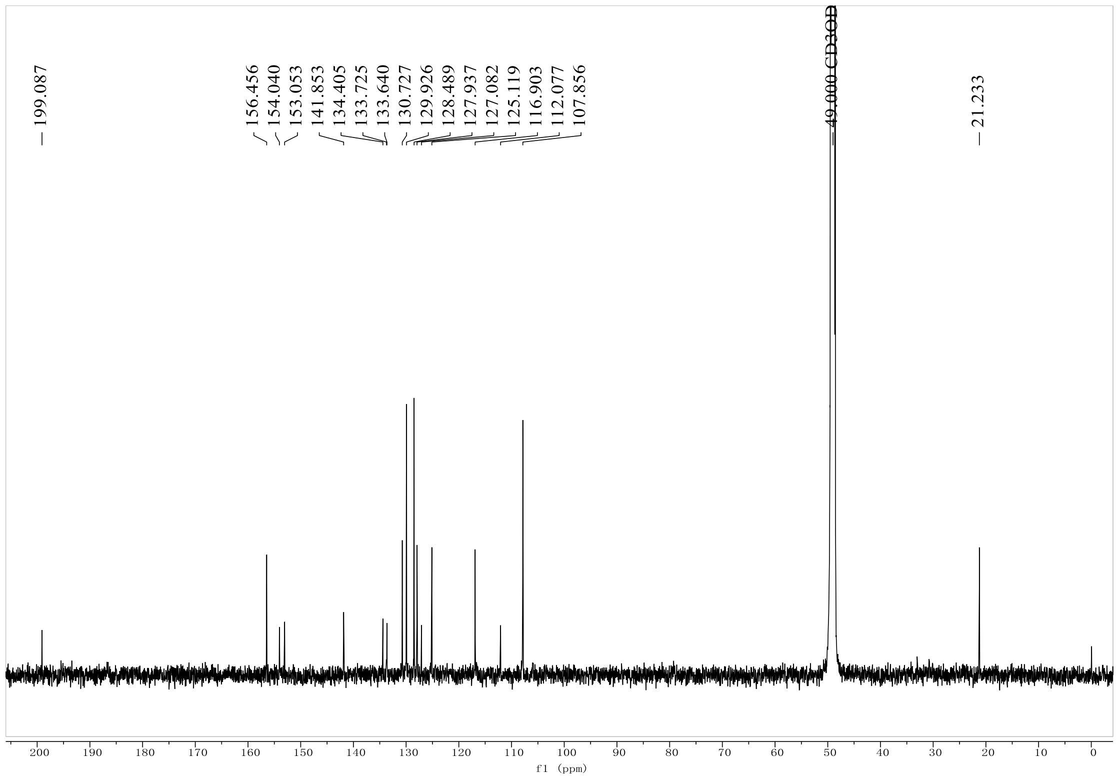

Figure S15. ^{13}C NMR spectrum of compound **3** (150 MHz, CD_3OD).

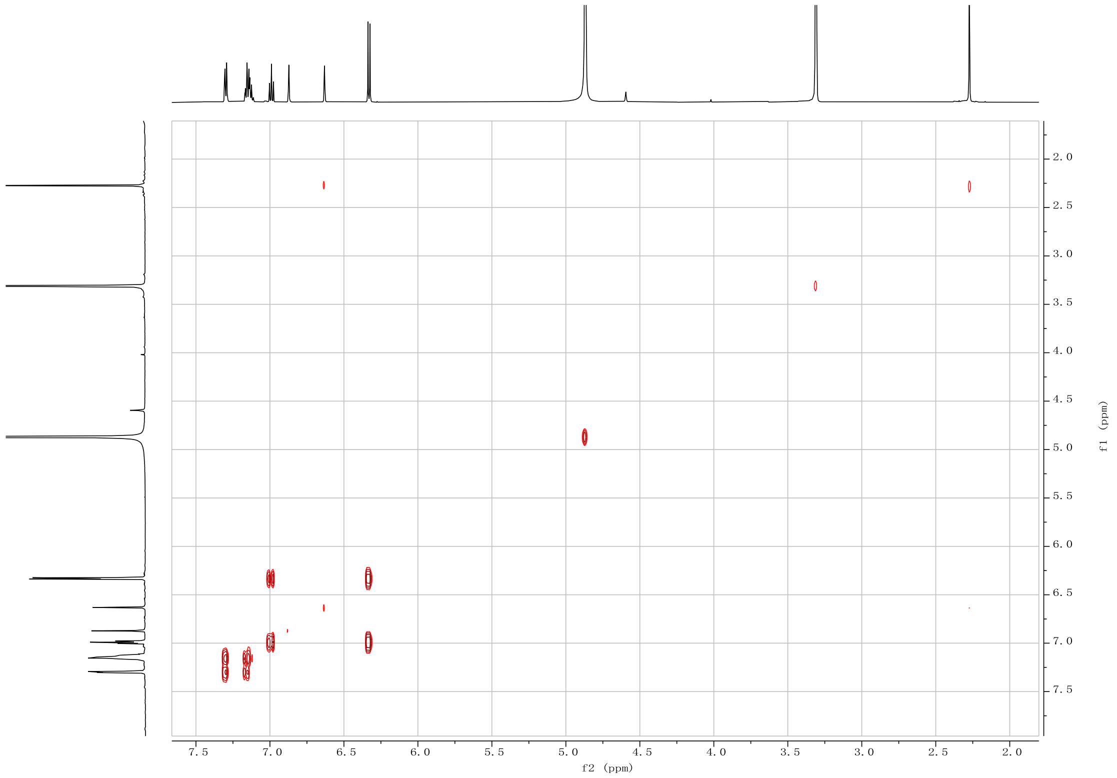

Figure S16. ^{1}H-^{1}H COSY spectrum of compound **3**.

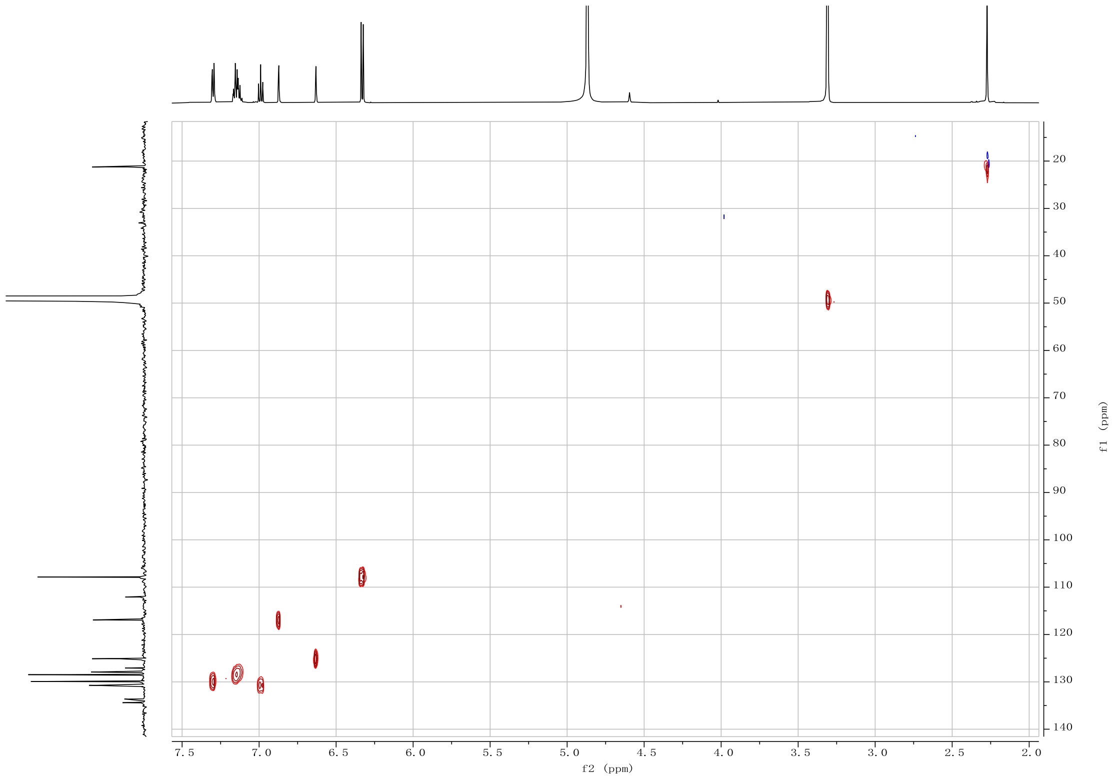

Figure S17. HSQC spectrum of compound **3**.

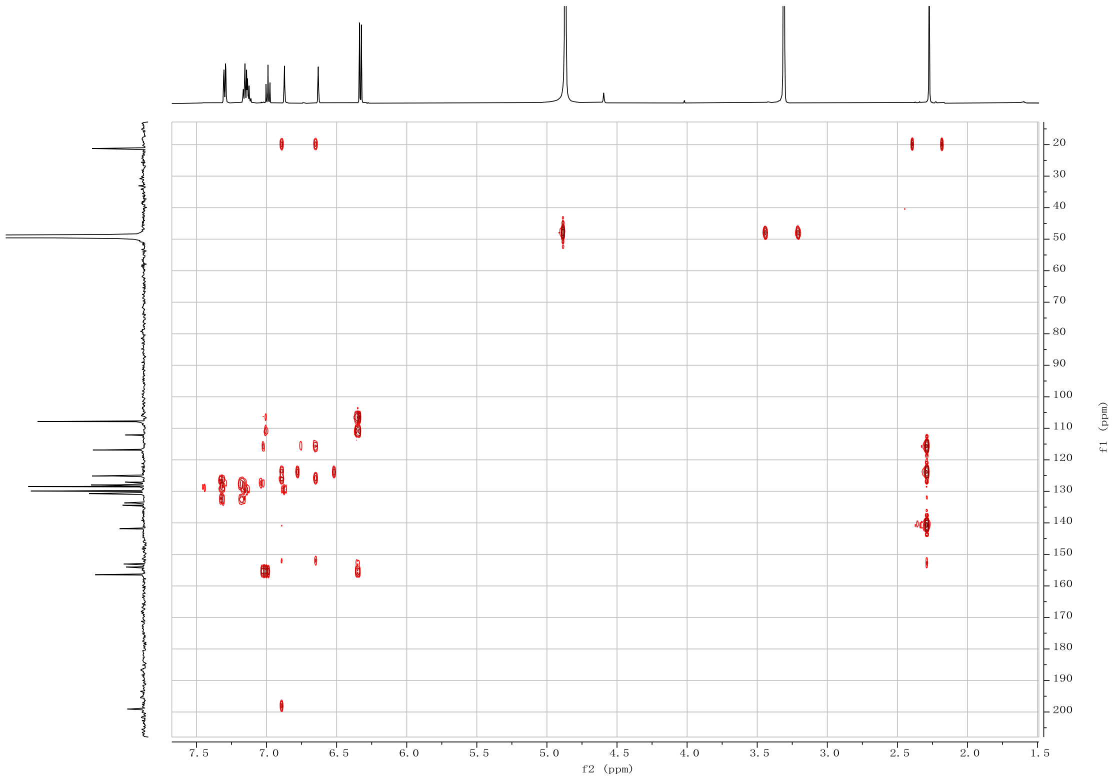

Figure S18. HMBC spectrum of compound **3**.

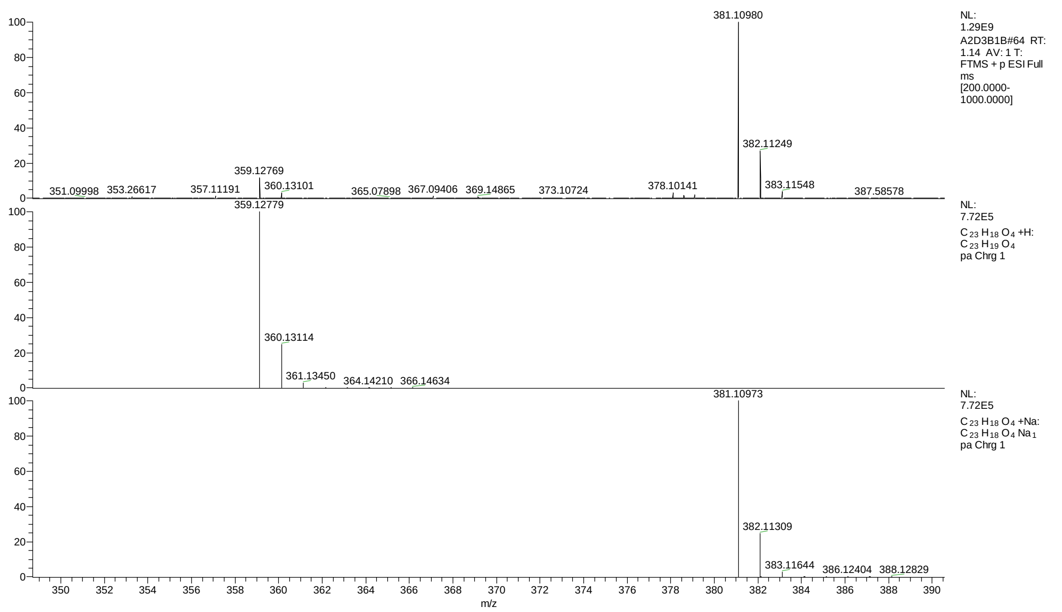

Figure S19. HRESIMS spectrum of compound **4**.

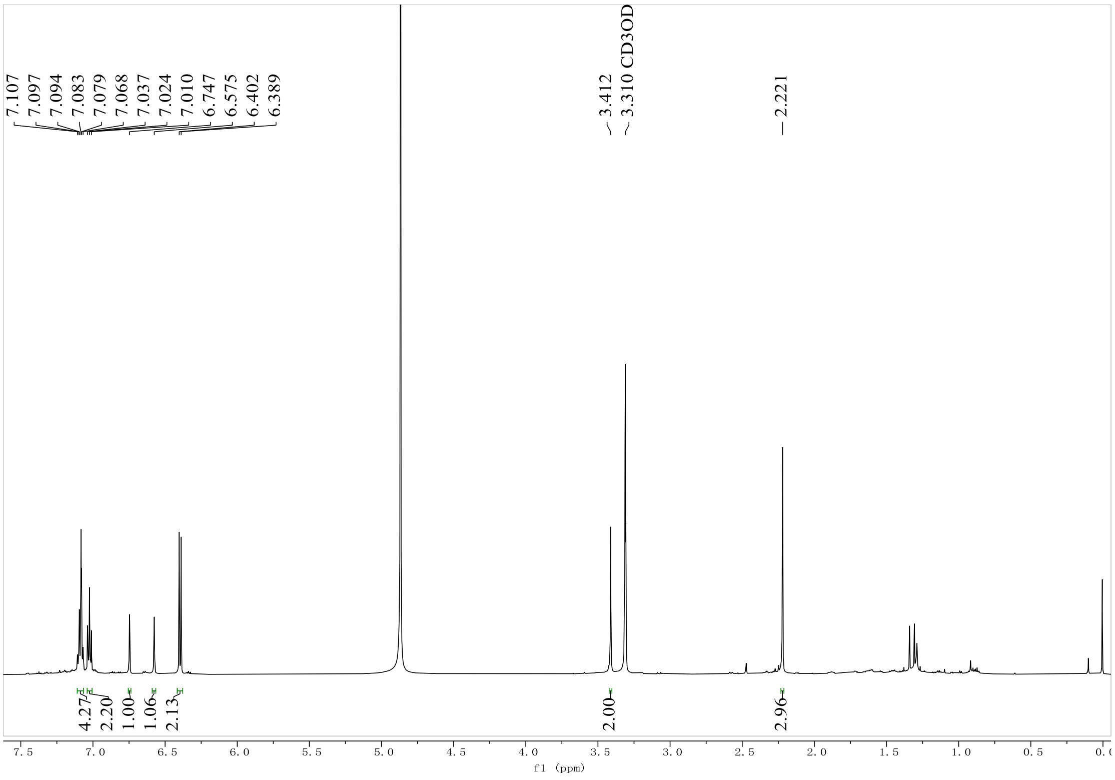

Figure S20. ^{1}H NMR spectrum of compound **4** (600 MHz, CD_3OD).

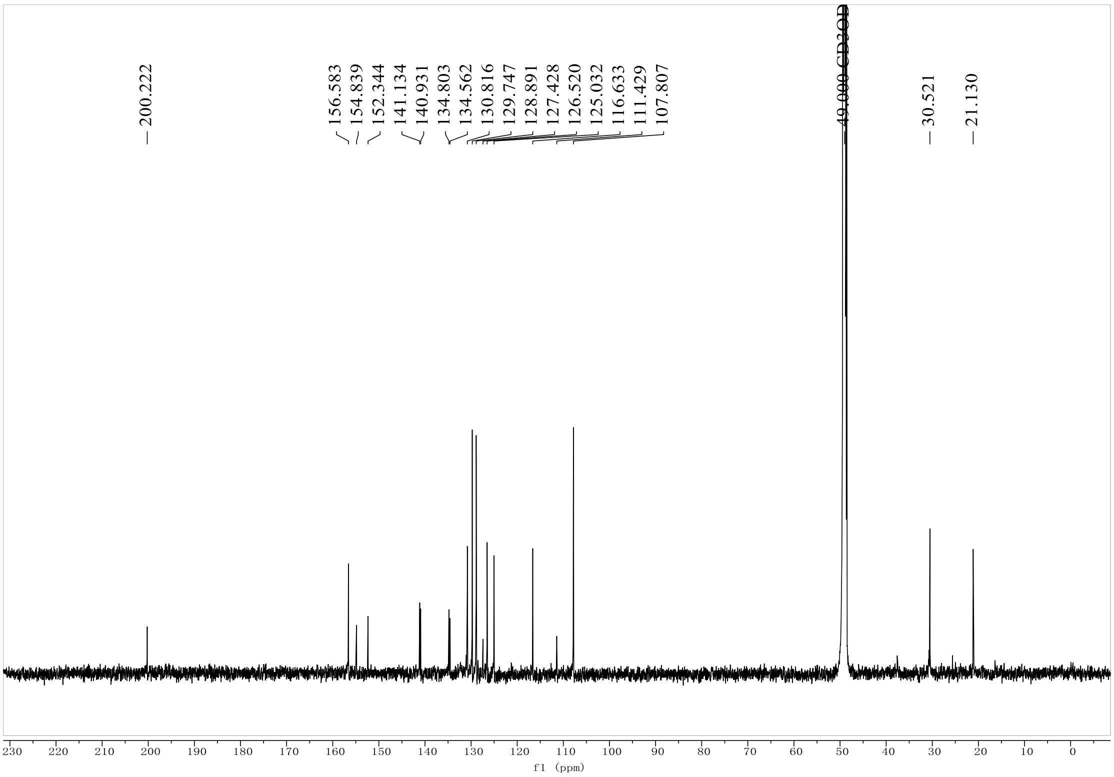

Figure S21. ^{13}C NMR spectrum of compound **4** (150 MHz, CD_3OD).

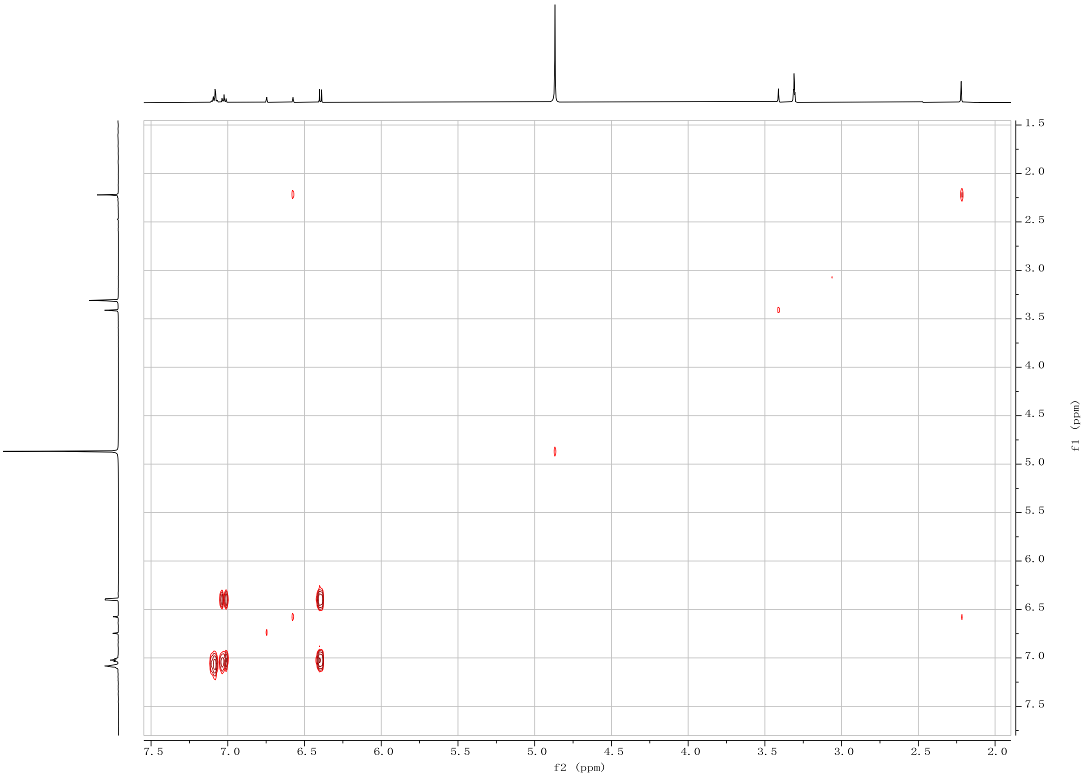

Figure S22. ^{1}H-^{1}H COSY spectrum of compound **4**.

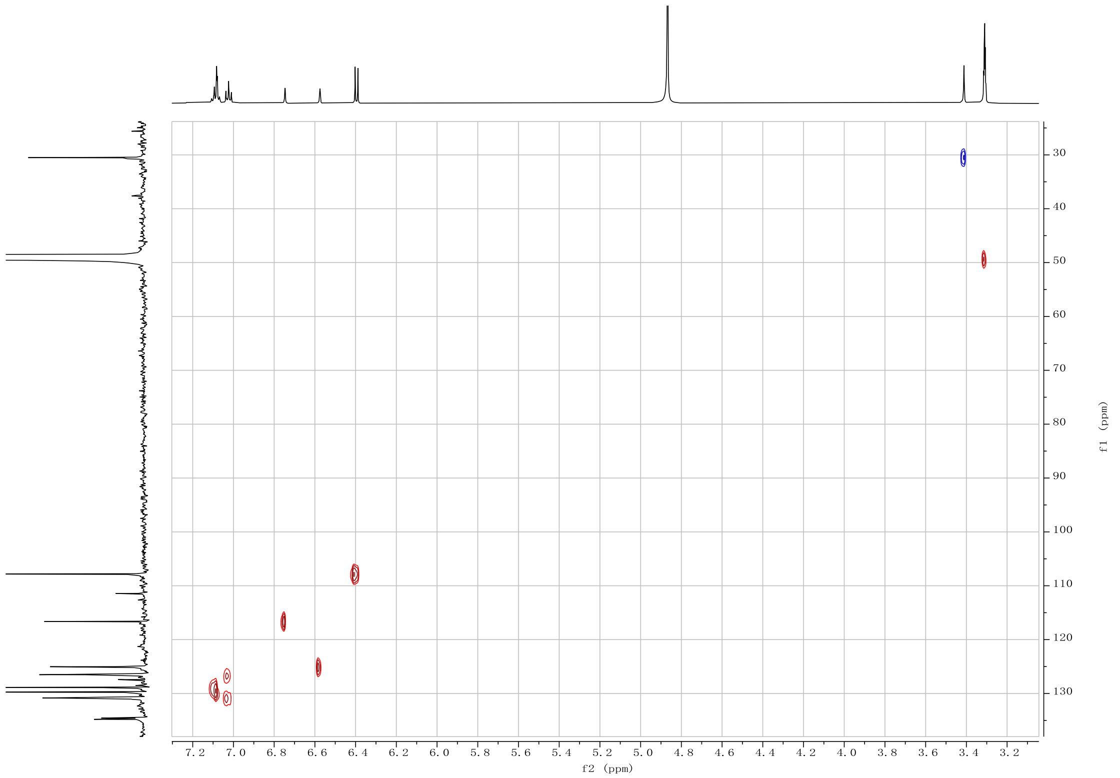

Figure S23. HSQC spectrum of compound **4**.

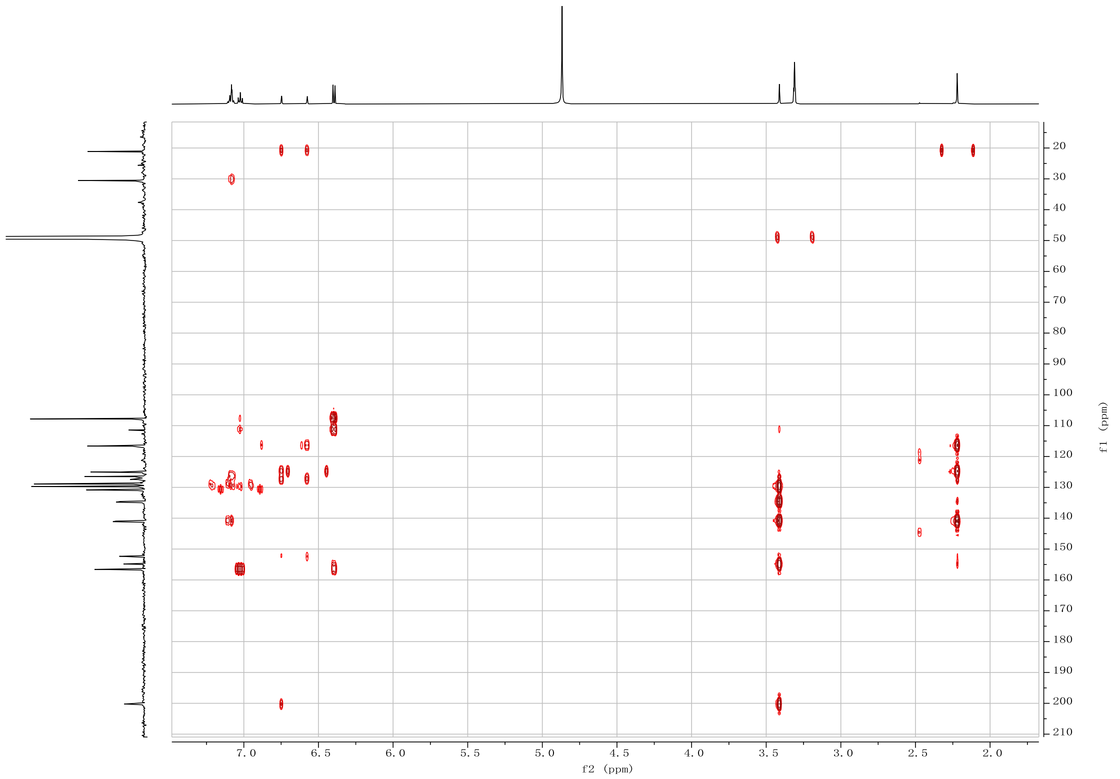

Figure S24. HMBC spectrum of compound **4**.

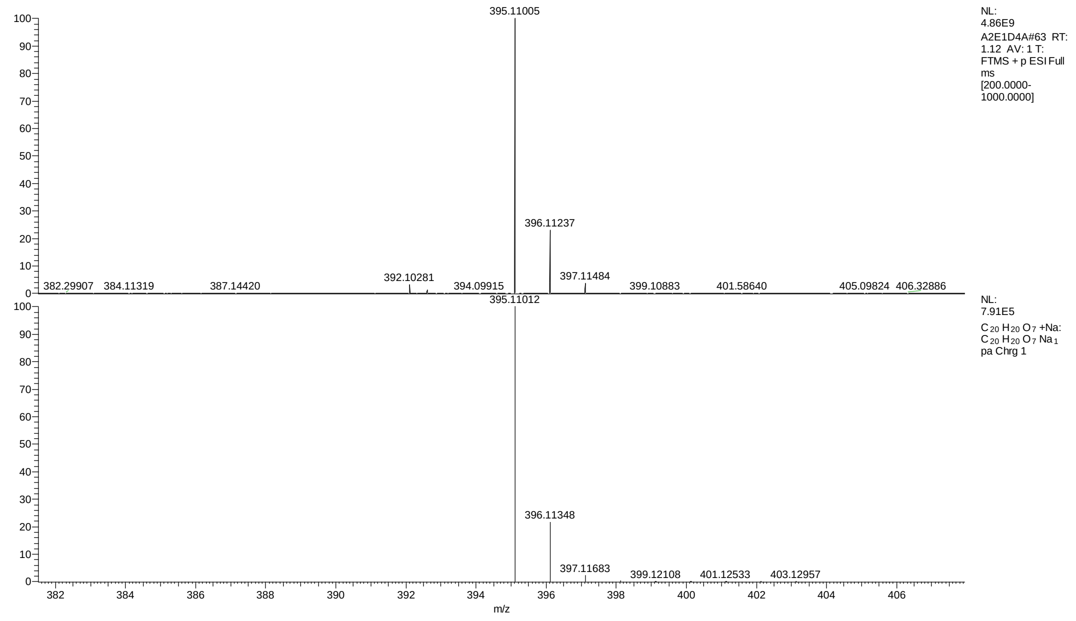

Figure S25. HRESIMS spectrum of compound **6**.

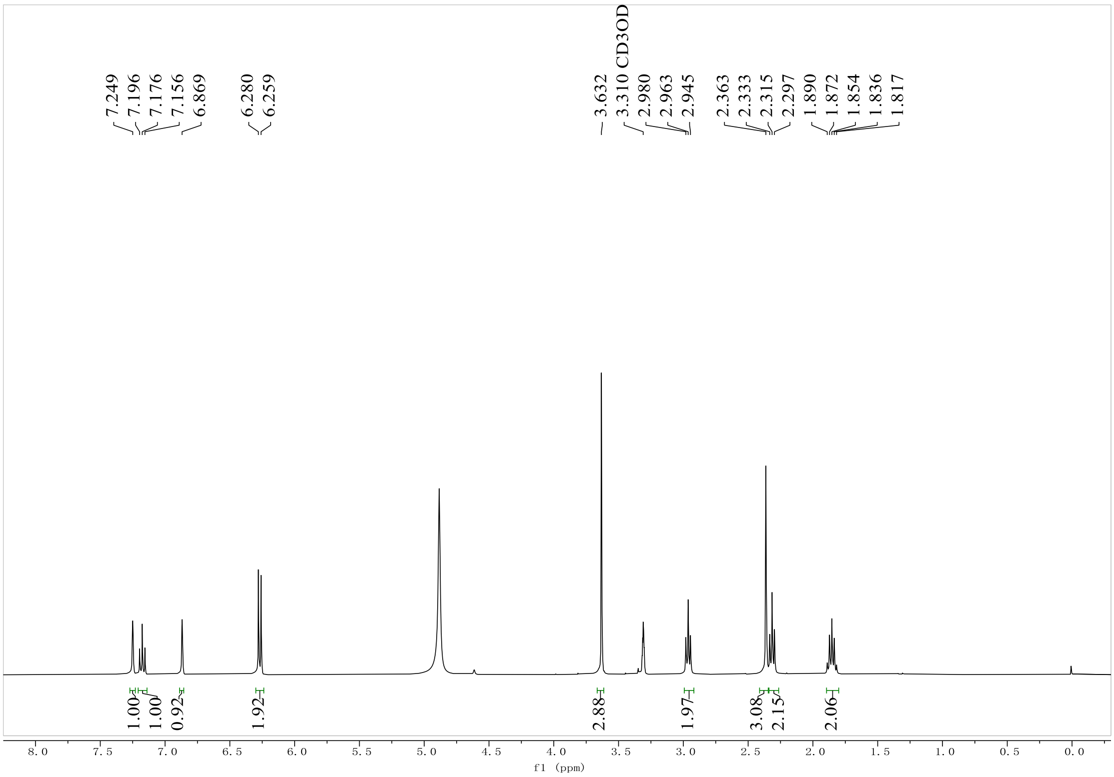

Figure S26. ^{1}H NMR spectrum of compound **6** (400 MHz, CD_3OD).

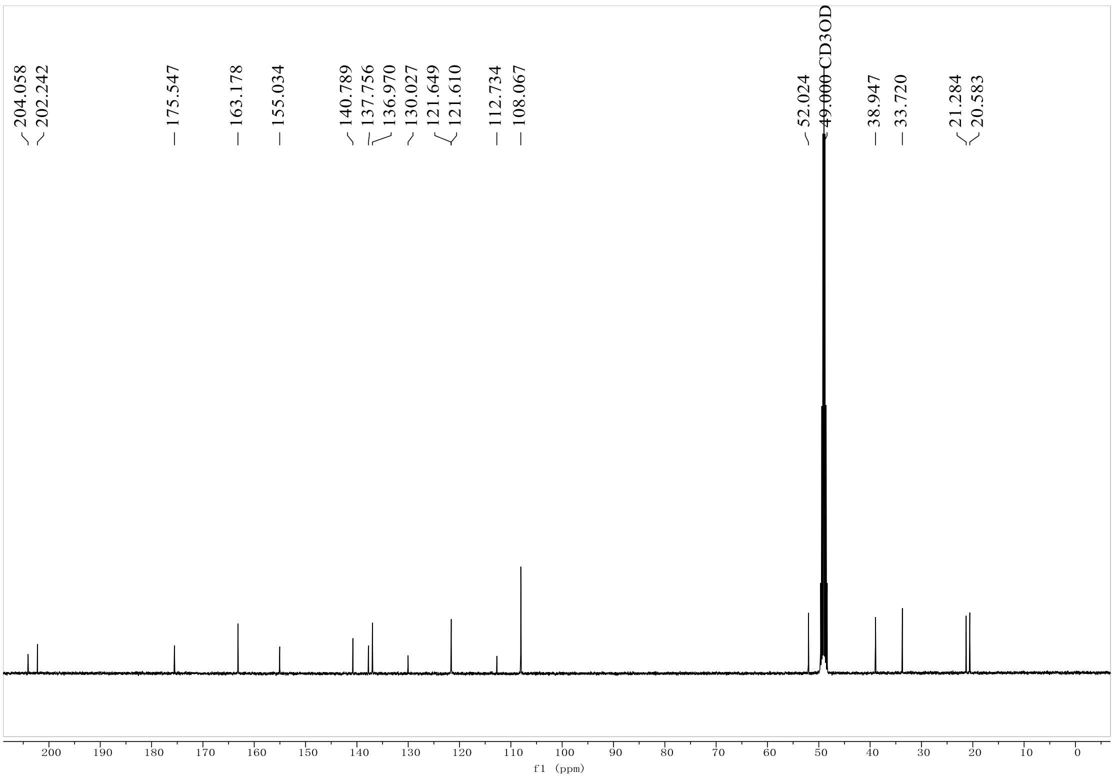

Figure S27. ^{13}C NMR spectrum of compound **6** (100 MHz, CD_3OD).

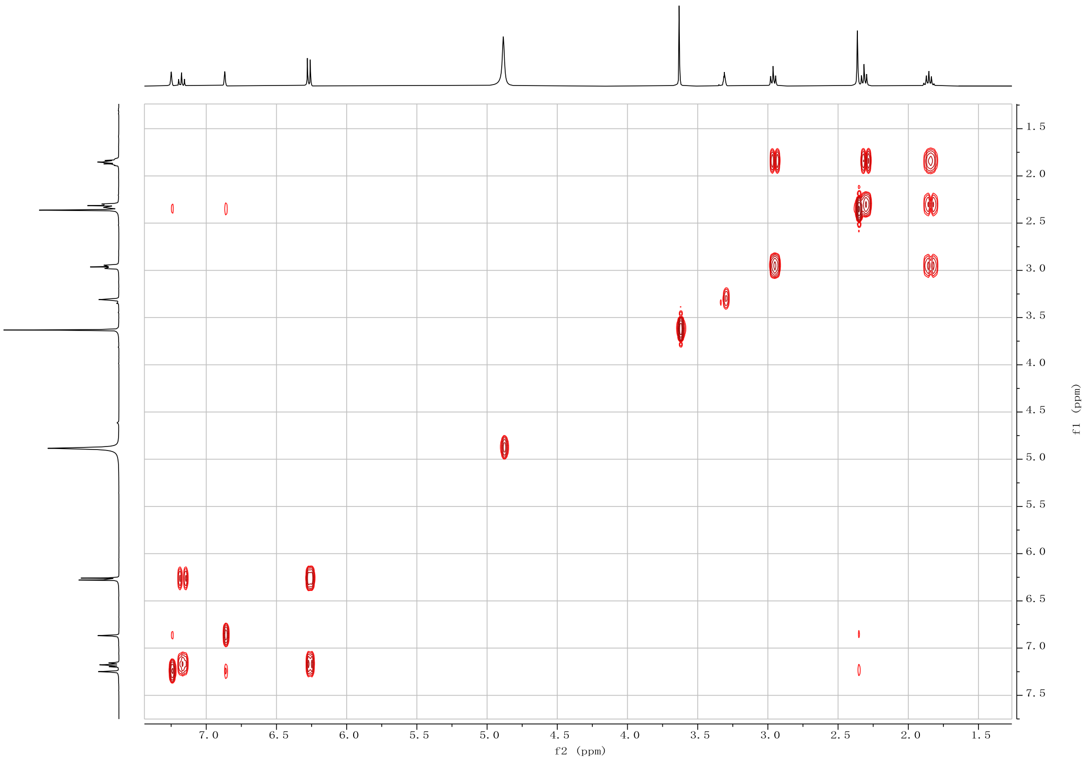

Figure S28. ^{1}H-^{1}H COSY spectrum of compound **6**.

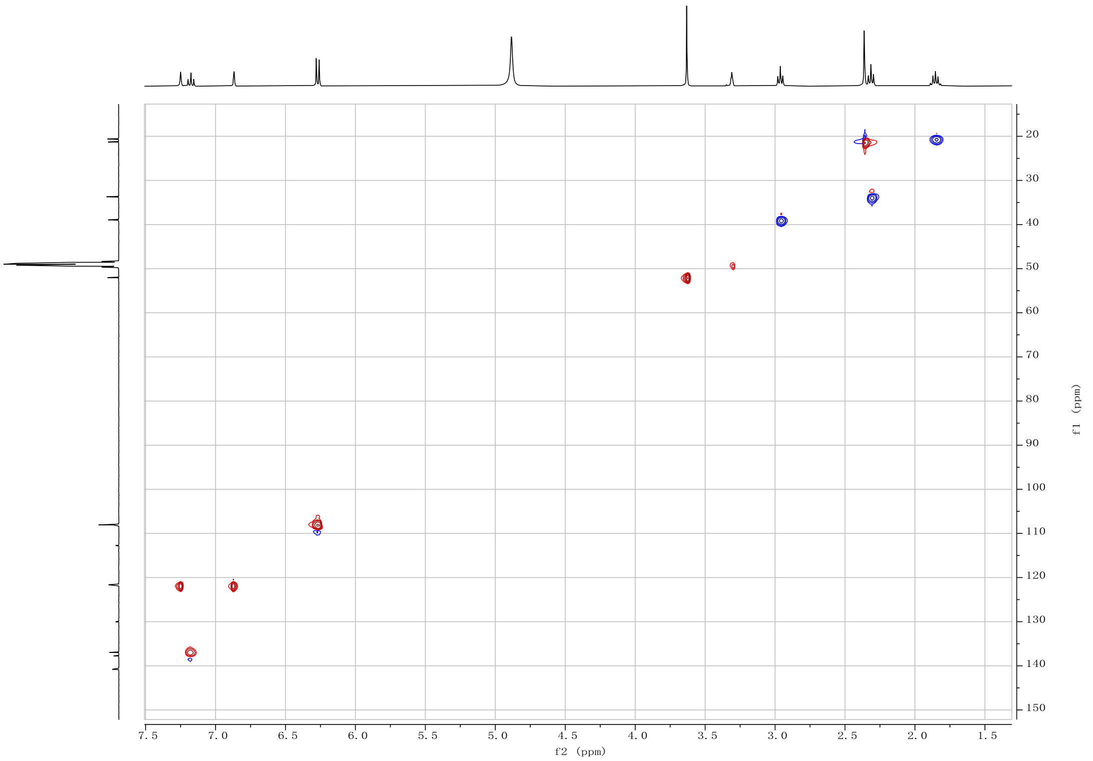

Figure S29. HSQC spectrum of compound **6**.

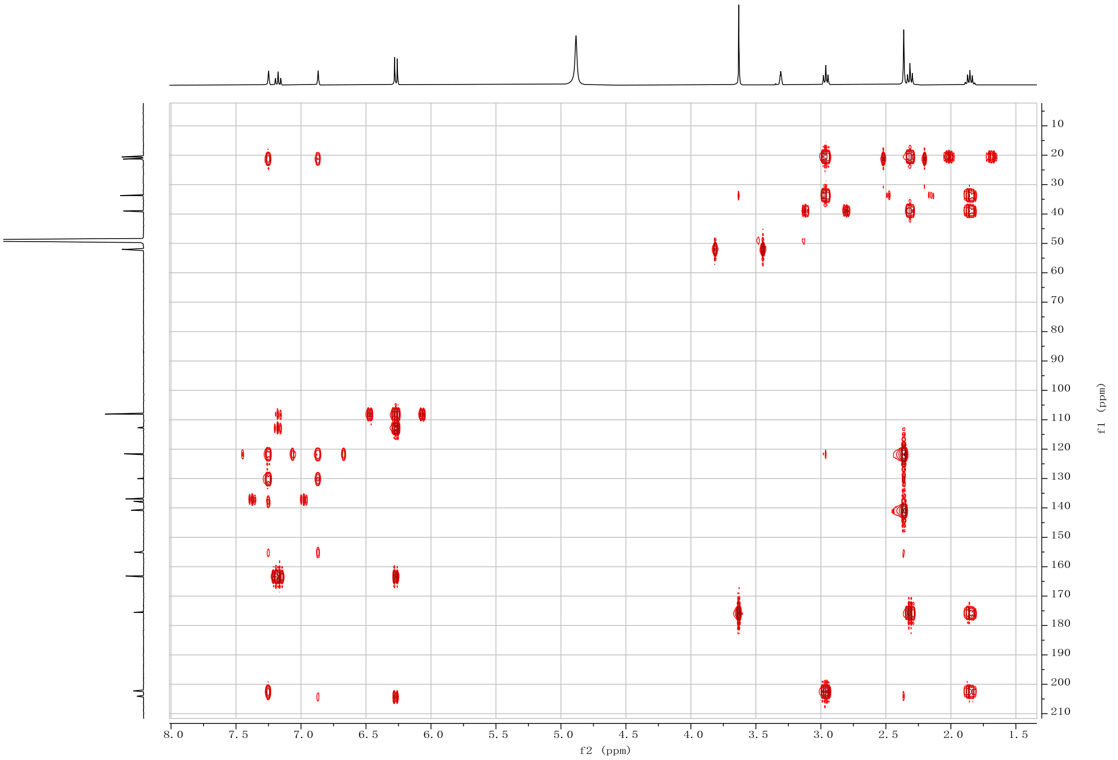

Figure S30. HMBC spectrum of compound **6**.

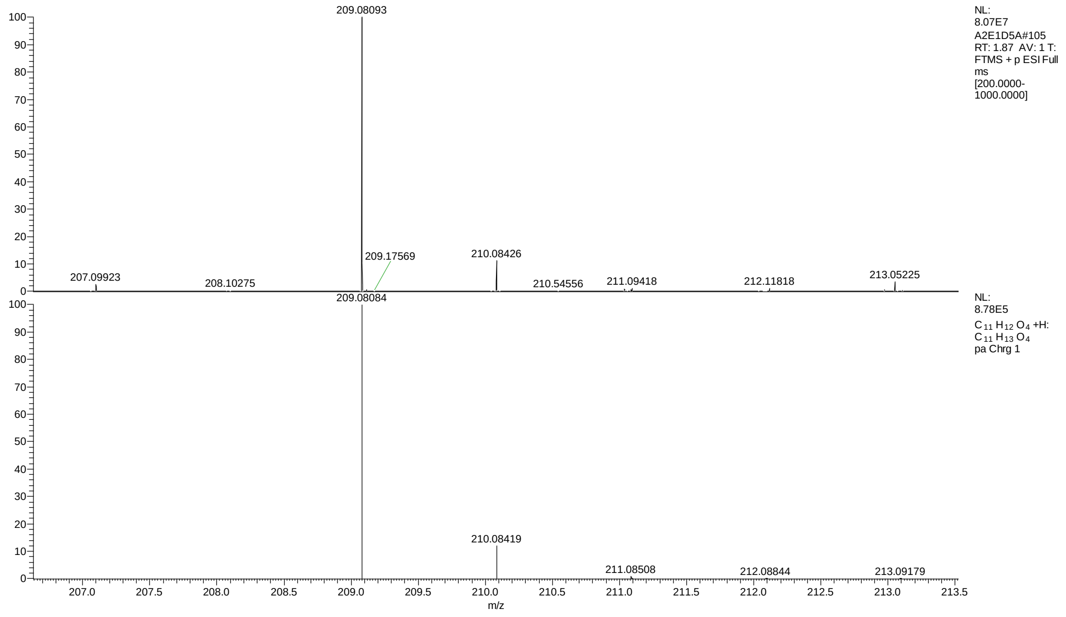

Figure S31. HRESIMS spectrum of compound **7**.

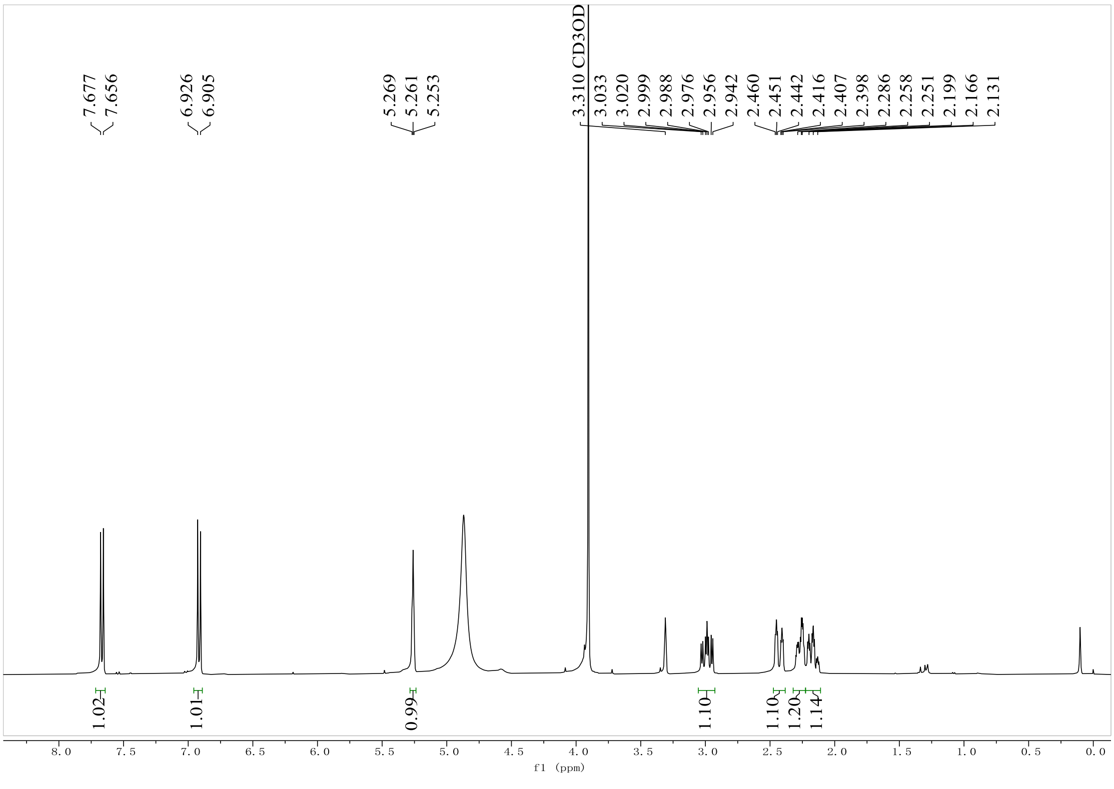

Figure S32. ^{1}H NMR spectrum of compound **7** (400 MHz, CD_3OD).

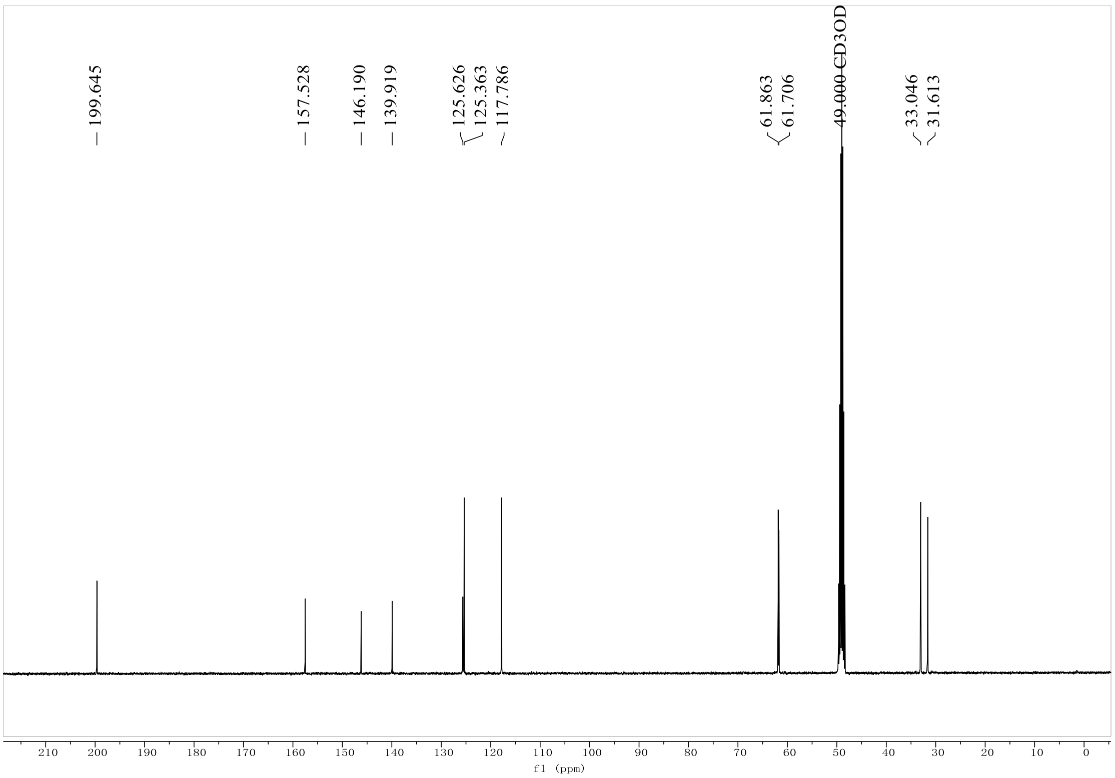

Figure S33. ^{13}C NMR spectrum of compound **7** (100 MHz, CD_3OD).

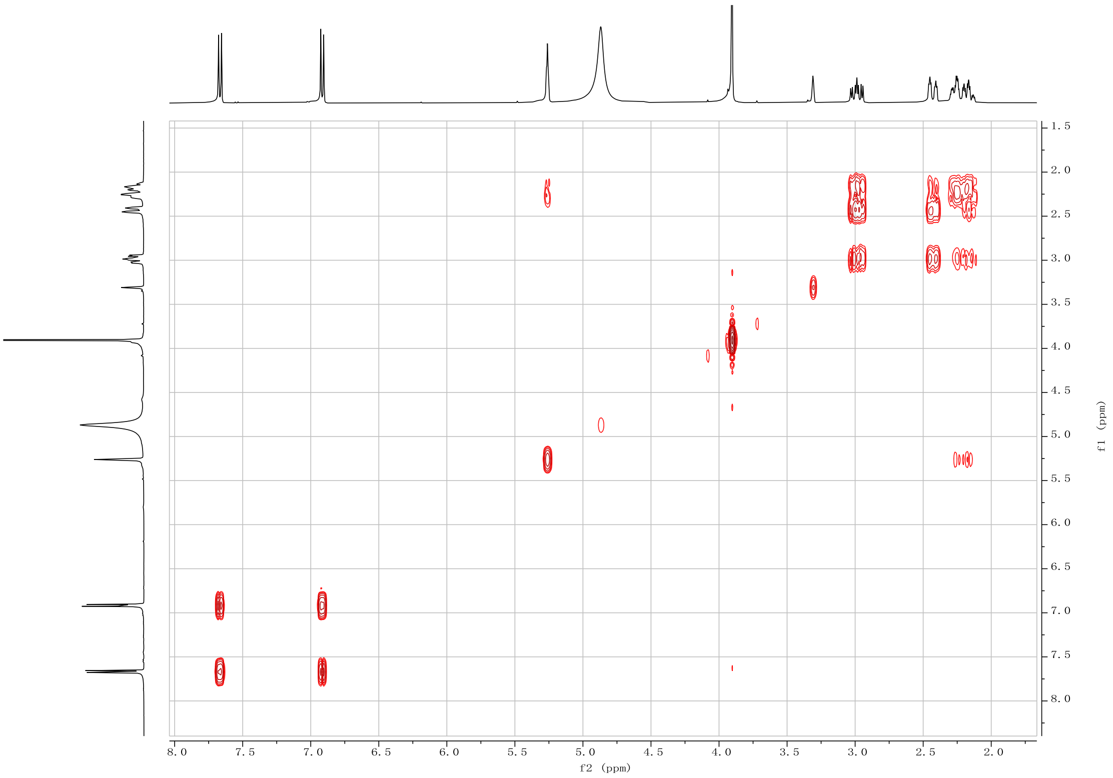

Figure S34. ^{1}H-^{1}H COSY spectrum of compound **7**.

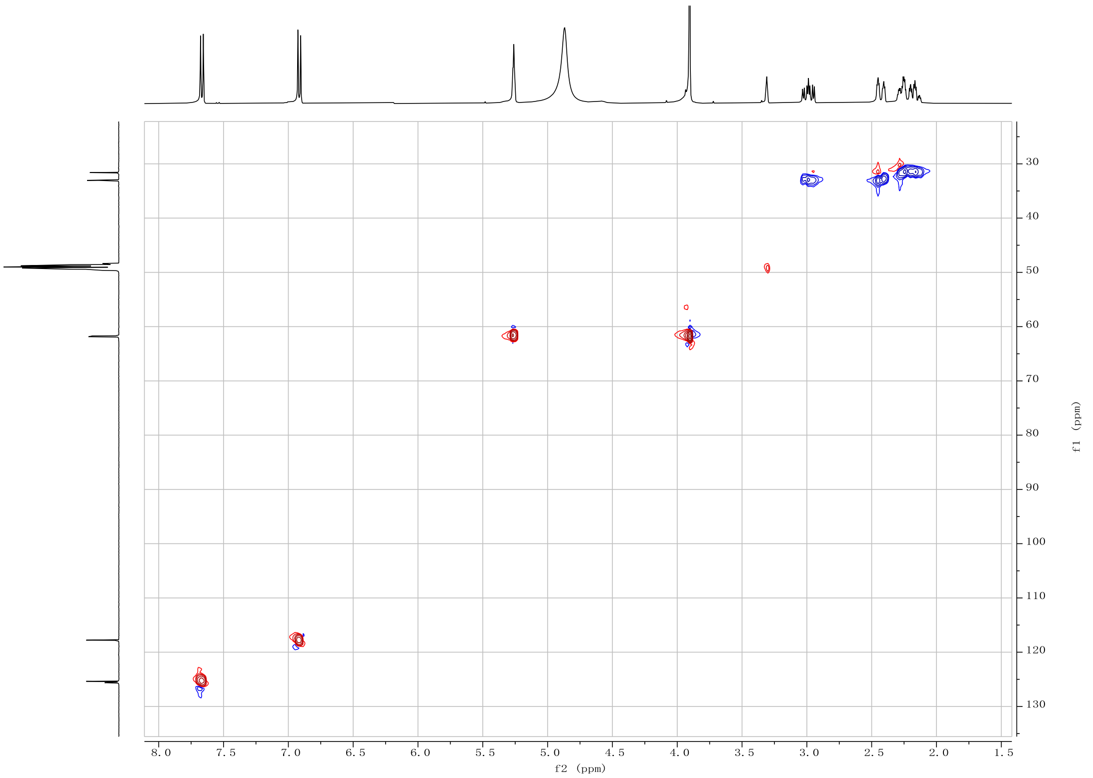

Figure S35. HSQC spectrum of compound **7**.

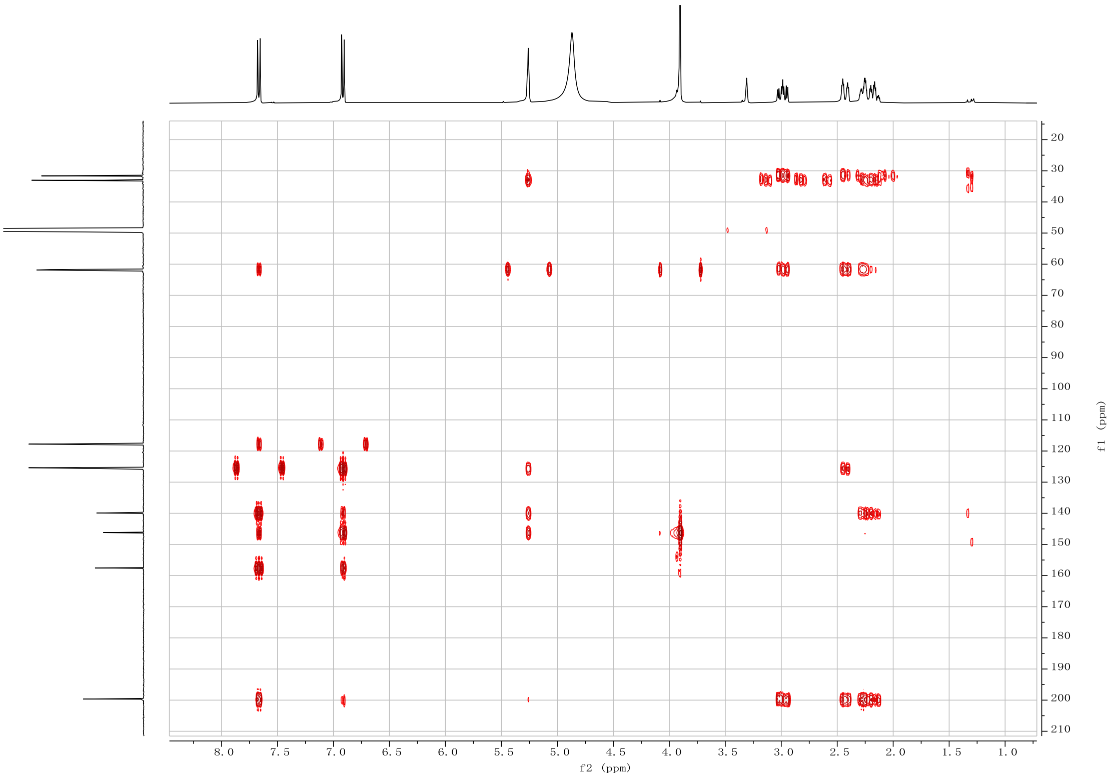

Figure S36. HMBC spectrum of compound **7**.

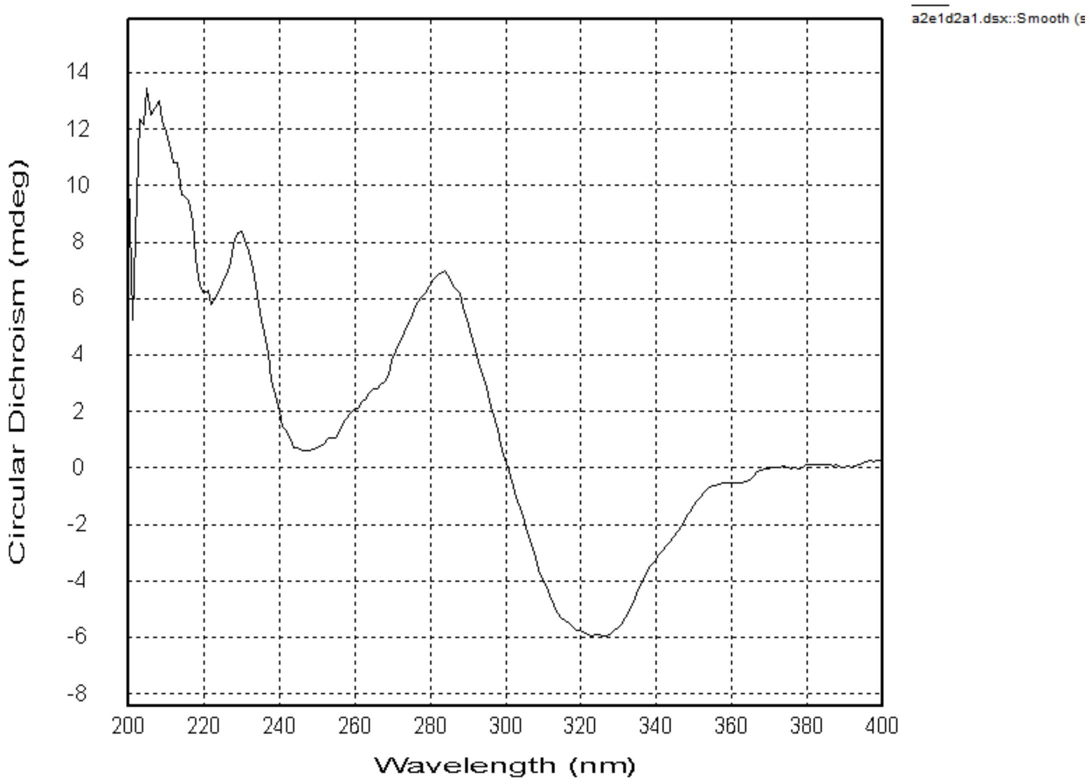

Figure S37. ECD spectrum of compound (+)-**7**.

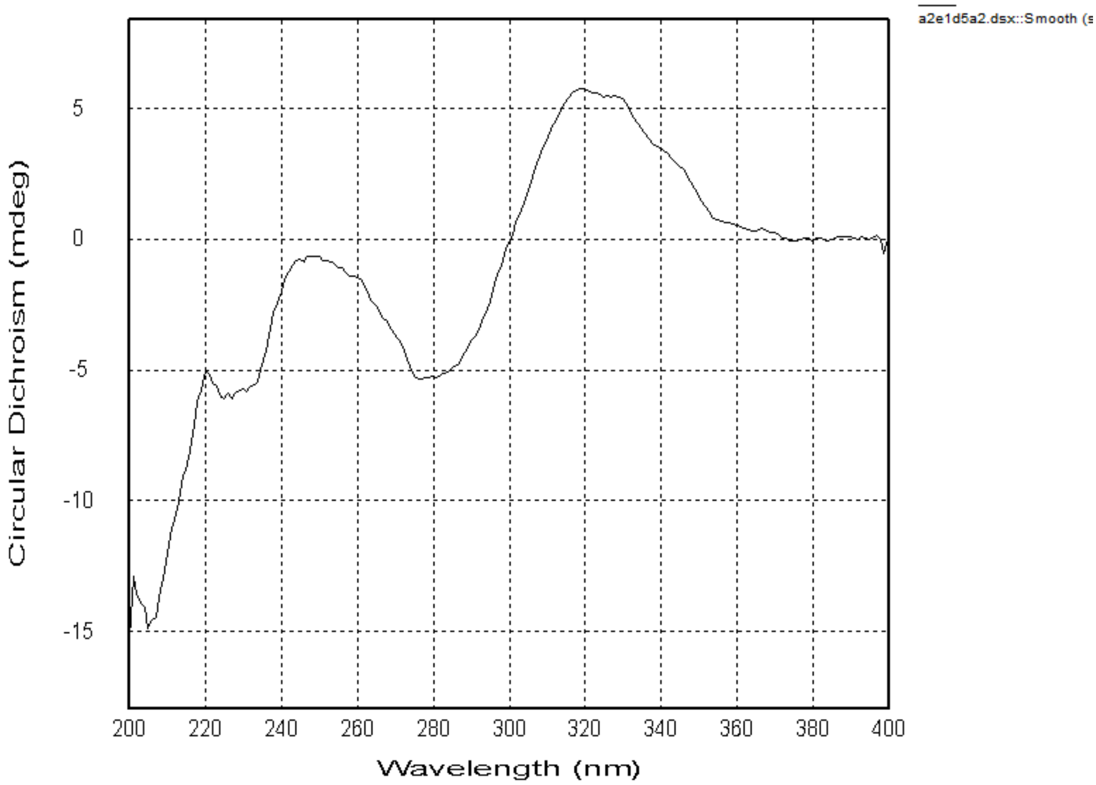

Figure S38. ECD spectrum of compound (−)-**7**.

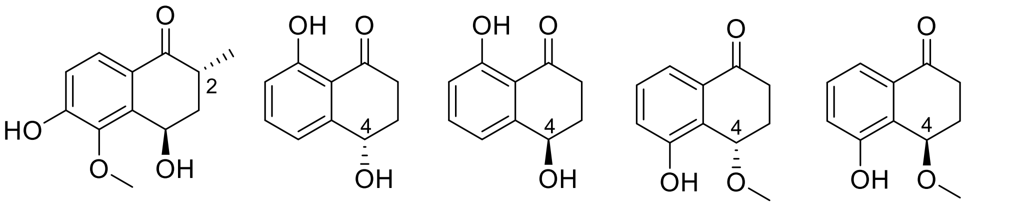

3,4-dihydro-4*β*,6-dihydroxy-5-methoxy-2*α*-methyl-1(2*H*)-naphthalenone

(4*S*)-4,8-dihydroxy-*α*-tetralone

(4*R*)-4,8-dihydroxy-*α*-tetralone

(4*S*)-5-hydroxy -4-methoxy-*α*-tetralone

(4*R*)-5-hydroxy -4-methoxy-*α*-tetralone

Figure S39. Structure of compounds 3,4-dihydro-4*β*,6-dihydroxy-5-methoxy-2*α*-methyl-1(2*H*)-naphthalenone, (4*S*)-4,8-dihydroxy-*α*-tetralone, (4*R*)-4,8-dihydroxy-*α*-tetralone, (4*S*)-5-hydroxy-4-methoxy-*α*-tetralone and (4*R*)- 5-hydroxy-4-methoxy-*α*-tetralone.
